# The impact of wearable continuous vital sign monitoring on deterioration detection and clinical outcomes in hospitalised patients: a systematic review and meta-analysis

**DOI:** 10.1186/s13054-021-03766-4

**Published:** 2021-09-28

**Authors:** Carlos Areia, Christopher Biggs, Mauro Santos, Neal Thurley, Stephen Gerry, Lionel Tarassenko, Peter Watkinson, Sarah Vollam

**Affiliations:** 1 Critical Care Research Group, Nuffield Department of Clinical Neurosciences, University of Oxford, Oxford, Oxfordshire UK; 2 Biomedical Research Centre, National Institute for Health Research, Oxford, UK; 3 Institute of Biomedical Engineering, Department of Engineering Science, University of Oxford, Oxford, Oxfordshire UK; 4 Bodleian Health Care Libraries, University of Oxford, Oxford, Oxfordshire UK; 5 Centre for Statistics in Medicine, Nuffield Department of Orthopaedics, Rheumatology and Musculoskeletal Sciences, University of Oxford, Oxford, UK; 6 Kadoorie Research Centre, Oxford University Hospitals NHS Foundation Trust, Oxford, UK

**Keywords:** Wearables, Deterioration detection, Vital signs, Wearable monitoring, Clinical outcomes, Hospital, ICU transfer

## Abstract

**Background:**

Timely recognition of the deteriorating inpatient remains challenging. Wearable monitoring systems (WMS) may augment current monitoring practices. However, there are many barriers to implementation in the hospital environment, and evidence describing the clinical impact of WMS on deterioration detection and patient outcome remains unclear.

**Objective:**

To assess the impact of vital-sign monitoring on detection of deterioration and related clinical outcomes in hospitalised patients using WMS, in comparison with standard care.

**Methods:**

A systematic search was conducted in August 2020 using MEDLINE, Embase, CINAHL, Cochrane Database of Systematic Reviews, CENTRAL, Health Technology Assessment databases and grey literature. Studies comparing the use of WMS against standard care for deterioration detection and related clinical outcomes in hospitalised patients were included. Deterioration related outcomes (primary) included unplanned intensive care admissions, rapid response team or cardiac arrest activation, total and major complications rate. Other clinical outcomes (secondary) included in-hospital mortality and hospital length of stay. Exploratory outcomes included alerting system parameters and clinical trial registry information.

**Results:**

Of 8706 citations, 10 studies with different designs met the inclusion criteria, of which 7 were included in the meta-analyses. Overall study quality was moderate. The meta-analysis indicated that the WMS, when compared with standard care, was not associated with significant reductions in intensive care transfers (risk ratio, RR 0.87; 95% confidence interval, CI 0.66–1.15), rapid response or cardiac arrest team activation (RR 0.84; 95% CI 0.69–1.01), total (RR 0.77; 95% CI 0.44–1.32) and major (RR 0.55; 95% CI 0.24–1.30) complications prevalence. There was also no statistically significant association with reduced mortality (RR 0.48; 95% CI 0.18–1.29) and hospital length of stay (mean difference, MD − 0.09; 95% CI − 0.43 to 0.44).

**Conclusion:**

This systematic review indicates that there is no current evidence that implementation of WMS impacts early deterioration detection and associated clinical outcomes, as differing design/quality of available studies and diversity of outcome measures make it difficult to reach a definite conclusion. Our narrative findings suggested that alarms should be adjusted to minimise false alarms and promote rapid clinical action in response to deterioration.

*PROSPERO Registration number*: CRD42020188633.

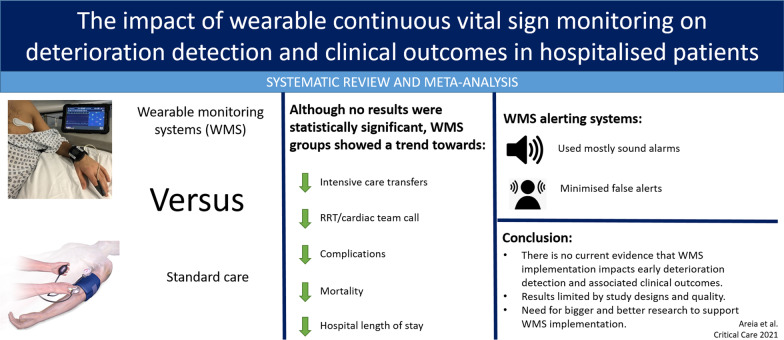

**Supplementary Information:**

The online version contains supplementary material available at 10.1186/s13054-021-03766-4.

## Introduction

### Background

Failure to recognise and act on physiological indicators of worsening illness in acute hospital wards is a generic problem that was recognised over a decade ago [1, 2] and may contribute to increased emergency intensive care unit (ICU) admissions and hospital mortality [1, 3]. In the United Kingdom, the use of physiological early warning scoring (EWS) systems (which measure "standard" vital signs such as pulse rate, respiratory rate, blood pressure, oxygen saturation, and temperature) is still common practice in general wards, together with a graded response such as referral for a senior review or increasing monitoring frequency [4]. This frequency of observations is generally guided by the clinical condition of the patient, and due to the requirement of manual physiological measurements, it can be time-consuming for healthcare professionals [5]. As a result, the optimal monitoring frequency is often not achieved [6], limiting the efficacy of intermittent monitoring systems dependent on the frequency of manual observations [7]. Furthermore, even when the ideal frequency is achieved, patients can deteriorate between observation sets [8]. Higher risk patients are often continuously monitored (for example in critical care), improving early detection of deterioration [5]. However, in the UK, continuous monitoring is not commonly used in the ward environment [9], although one study suggests it may be feasible and cost-effective in surgical wards [10], with the potential to improve patient outcomes when compared to intermittent monitoring [8].

Despite the potential to promote earlier detection of deterioration, limitations in continuous vital sign monitoring technology can pose a barrier to implementation [5], such as restriction of patient mobility and independence due to wires and static devices [9, 11]. In response to this need, commercially available wearable monitoring devices are evolving rapidly [12]. Wearable devices may provide an alternative to static wired continuous monitors and offer a bridge between bedside wired monitoring and intermittent manual measurements. This development has the potential to promote patients’ mobility and comfort while reducing nursing time and improving the early detection of abnormal physiological parameters [13].

A recent meta-analysis assessed the impact of multi-parameter continuous non-invasive monitoring in hospital wards, including wired static devices, suggesting a 39% decreased mortality risk in monitored patients compared to those receiving standard care (intermittent manual observations), it also suggested a trend of reduced intensive care unit (ICU) transfer, rapid response team (RRT) activation and hospital length of stay (LoS) [14]. The validation, feasibility, costs and clinical outcomes of 13 different wearable devices were assessed in another systematic review, which demonstrated that the majority of studies were still at the validation and feasibility phases [15], emphasising the lack of evidence assessing the impact on economic and clinical outcomes as there is still uncertainty around the impact of wearable monitoring systems (WMS) in the hospital environment, hindering its implementation and clinical use [16]. Our review focused on these wearable monitoring devices and/or systems implemented inside the hospital (inclusive of all specialities, acuity and ages).

### Objective

The objective of this systematic review and meta-analysis was to assess the impact of vital sign monitoring on the detection of physiological deterioration and related clinical outcomes of hospitalised patients using wearable monitoring systems in comparison with standard care.

## Methods

This systematic review was registered with the International Prospective Register of Systematic Reviews (PROSPERO) on the 10th July 2020, registration number CRD42020188633 [17]. This review was reported following the Preferred Reporting Items for Systematic Reviews and Meta-Analyses (PRISMA) checklist (Additional file 1: Appendix 1) [18]. The full systematic review protocol was published prospectively [19].

### Outcomes

#### Primary outcomes

This study aimed to compare the impact of wearable monitoring systems on deterioration detection and related clinical outcomes metrics, in comparison with standard care. A variety of outcomes related to deterioration detection were anticipated, and therefore searches were not limited by outcome. Any outcome related to the detection of deterioration was included as a primary outcome for this review.

A variety of complications related to clinical deterioration were reported and included in the meta-analysis, from minor (for example fainting, or shortness of breath) to major (such as life-threatening events). A separate analysis was then conducted for the studies separately reporting major complications; the Clavien-Dindo system [20] was applied to postoperative complications in the included studies. This system grades complications from I (deviation from usual recovery not requiring intervention) to V (patient death). To be included in our major complication meta-analysis we included complications defined by a Clavien-Dindo grade of > II (8,18). Patient death and ICU transfer were not included in this analysis and were assessed separately.

Outcomes reported in less than 3 studies were not included in the meta-analysis and were instead narratively described such as time to antibiotic administration in case of sepsis and number of the National Early Warning Score (NEWS) measurements.

The primary outcomes reported in the meta-analysis included ICU transfers, rapid response or cardiac arrest activation, and complications. Time to antibiotic administration in case of sepsis was narratively reported.

#### Secondary outcomes

Secondary outcomes in the meta-analysis included in-hospital mortality and hospital length of stay. Further secondary patient outcomes were reported in the narrative analysis, such as 30-day readmission rates and time to post-operative mobilisation.

#### Exploratory outcomes

Exploratory outcomes included the alerting systems used, implementation and iterations in clinical practice. This included type of early warning score, alarm thresholds used for each vital sign or overall EWS and other relevant alarm parameters/information, where available.

Clinical trial registry searches were also conducted. For included studies that were registered, a comparison was made between the details in registration and report of the study. Registered studies eligible for inclusion but without published results were also narratively reported.

### Eligibility criteria

#### Population and interventions

Complete inclusion and exclusion criteria are available in the published protocol [19]. We included any studies conducted in hospitalised patients, excluding studies conducted in healthy volunteers or non-hospitalised patients. Age was not a restriction for inclusion, however one of the included studies monitored fetal heart rate, and was not included in the meta-analysis because of the differing population and outcomes analysed.

Studies were eligible for inclusion if they used a wearable monitoring system (with or without standard care) in comparison with standard care. Included WMS required to monitor at least one vital sign (heart rate, respiratory rate, temperature, blood pressure or oxygen saturation), sampled continuously at a high rate (e.g. under a minute) or low rate (e.g. every 5 min) and where measurements did not require frequent manual input from clinical staff. For comparator we considered any type of standard care for vital sign monitoring, as defined in the study protocol [19].

#### Study types

Studies with the following designs were considered for inclusion: randomised controlled trials (RCTs), cluster RCTs, interventional studies, observational studies (including case–control and before-after studies), and pilot studies. Retrospective studies that complied with the proposed outcomes and eligibility criteria, and unpublished (grey) literature, were also considered.

### Literature search and selection of studies

Search terms were designed by a medical librarian with field expertise (NT). Relevant articles up to 27th August 2020 were identified through electronic searches on MEDLINE Ovid (including Epub Ahead of Print and In-Process & Other Non-Indexed Citations), Embase (Ovid), CINAHL (EBSCO) and Cochrane Database of Systematic Reviews (Cochrane Library, Wiley), Cochrane Central Register of Controlled Trials (CENTRAL) (Cochrane Library, Wiley) and Health Technology Assessment (HTA) database via https://www.crd.york.ac.uk/CRDWeb/. Full search strategy and terms/combinations used in each database can be found in Additional file 2: Appendix 2.

Clinical trials and prospective studies registered up to 10th September 2020 in ClinicalTrials.gov via https://clinicaltrials.gov/ and ISRCTN via https://www.isrctn.com/ were also identified. Search details in Additional file 3: Appendix 3.

Searches of unpublished grey literature and pre-print servers were also conducted manually (details in Additional file 4: Appendix 4) and additional studies published in these servers up to 16th December 2020 were identified.

Titles and abstracts of all potentially relevant articles were independently reviewed for possible inclusion by two authors (CA, CB). The full text of any citation considered potentially relevant by any reviewer was retrieved. The degree of interrater agreement for study selection was determined by using kappa, with standard definitions for poor (< 0.20), fair (0.21–0.40), moderate (0.41–0.60), good (0.61–0.80), and very good (0.81–1.00) agreement [21, 22]. The included abstracts full-texts were assessed for eligibility and disagreements resolved by discussion between the 2 review authors; if no agreement could be reached, a third author was consulted (SV). The full selection process is outlined in the published protocol [19].

### Data collection and extraction

Two reviewers (CA and CB) extracted the data independently from the included studies. Disagreements were resolved by discussion between the 2 review authors. When required, this was also discussed with a third author (MS) and a statistician (SG).

The following data were extracted for each study, where available: author list, country, date published, registration number, aim, design, setting and population, recruitment start and end dates, ethical approval and informed consent information, eligibility criteria, intervention description, included devices, period of device wear, vital signs measured by devices, frequency of wearable data availability, comparator type, EWS and frequency of manual measurements, sample size, demographics (e.g. age, gender, BMI, etc.), other clinical characteristics (e.g. type of admission, American Society of Anaesthesiologists, ASA, score, etc.), deterioration detection and related clinical outcomes summary data, total/median monitoring hours, alerting system information (e.g. thresholds and alarms description), study limitations, device FDA/CE mark information, funding and conflict of interest information.

### Risk of bias of individual studies

Four tools, selected based on study design, were used to assess risk of bias. For randomised controlled trials the Cochrane risk of bias tool (RoB2) was used [23]; for non-randomised studies, the Newcastle Ottawa Scale (NOS) [24] and the “Risk Of Bias In Non-randomised Studies—of Interventions” (ROBINS-I) were applied [25]; and, in addition, the Mixed Methods Appraisal tool (MMAT) [26] was used for all studies. This was a change from the original protocol [19] as the Jadad scale was replaced by the ROBINS-I for assessment of non-randomised studies, as we found it more comparable with the ROB2 tool used for included RCTs. Two reviewers (CA and CB) independently appraised each study and disagreements were solved by discussion until consensus was reached with a third reviewer (SV).

### Data analysis

All outcomes with results from at least three studies were considered for the meta-analysis. Outcomes with data from less than three studies were not included in the meta-analysis but reported in a narrative synthesis.

#### Data preparation and meta-analysis

Review Manager 5.4.1 (The Cochrane Collaboration, Oxford, England [27]) was used to calculate pooled risk ratios (RRs) for dichotomous outcomes and pooled weighted mean differences for continuous outcomes, and respective 95% confidence intervals (CIs) [28]. Continuous variables are expressed as mean (SD). Due to differences in design between included studies, we used random-effects meta-analysis and the TAU2 statistic, and respective significance level was calculated [28]. We assessed heterogeneity among trials by using I^2^ (the percentage of total variability across studies attributable to heterogeneity rather than to chance) and used published guidelines for interpretation [27].

One before-and-after study compared the WMS group with a before period in the same unit and a different unit (both before and during) [29]. For the meta-analysis, we limited data to that reported from the same unit to minimising confounding. Outcomes for this study were also presented per 1000 discharges. As the authors provided the total number of discharges, the actual event numbers were calculated for inclusion in our analysis [29]. Similarly, another included study presented the hospital length of stay (LoS) in hours [30]; this was converted to days for the analysis. In a further study, the authors presented LoS in median (Interquartile range, IQR) format, which was converted to mean (standard deviation, SD) format. A normal distribution of the values was assumed to make this conversion, as per Cochrane guidance [31].

Finally, in one study complication data was presented as the number of events rather than the number of patients suffering a complication [32, 33]. A formal data request to the principal investigator was made to acquire the data in the correct format, and this was used in the meta-analysis.

#### Narrative analysis

Alerting thresholds, methods and other alarm information was extracted from the included studies, where available, and narratively reported. For analysis of study registration, the proportion of registered studies that were published, and both the dates of trial registration and publication of results were reported. We also explored registered versus published primary and secondary outcomes. Principal Investigators for the included and registered studies were contacted for further information, as required.

#### Body of evidence summary

A body of evidence summary is provided in Additional file 5: Appendix 5, using the GRADEpro software [34].

## Results

### Study selection

**Fig. 1:**
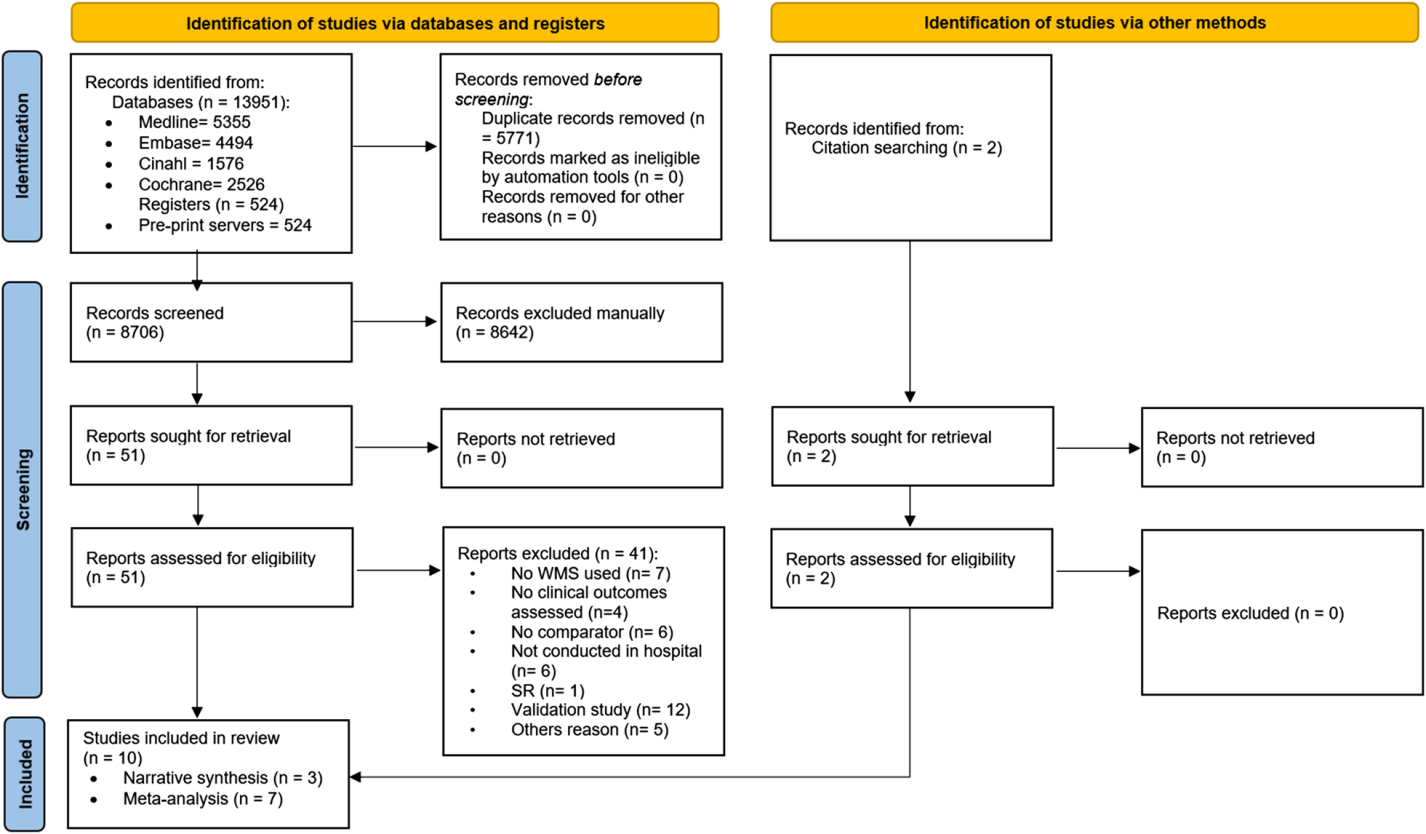
PRISMA Flow diagram. WMS: Wearable Monitoring System

After removal of duplicates, 8706 studies were identified. Following title and abstract review 51 full texts remained, of which 10 met the inclusion criteria (Fig. 1). Four studies appeared to meet the inclusion criteria but were excluded at full-text review: two studies were excluded for not reporting a subset of their analysis for the patients using the WMS [35, 36]; one was excluded after confirming with the author that the device was not wearable at the time of the study [37], and another did not have a comparator group [38]. A total of 4433 patients were included in these studies.

### Study characteristics

#### Devices

A variety of devices were used in the studies included in this review: four studies used the VisiMobile (Sotera Visi Mobile, San Diego, California) [29, 39–41], with two studies also adding the HealthPatch (Vital Connect, Campbell, CA, USA) [40, 41]; two studies used Sensium Vitals (Sensium, Abingdon, United Kingdom) [32, 33]. The remaining four studies used different devices, including the Patient Status Engine (Isansys Lifecare Ltd.) [30], the Auricall monitoring system [42], the Avant-4100 (Nonin) [43] and the Monica Novii wireless patch system (General Electric Company, Milwaukee, WI) [44].

Of the ten studies identified, seven were included in the meta-analysis with a total of 4127 patients. These included two RCTs [30, 33], one cluster RCT [32], and four before-and-after observational studies [29, 39, 42, 43]. Three further studies (RCTs) were included in the narrative synthesis [40, 41, 44], including a total of 306 further patients. Details of the included studies are presented in Tables 1 and 2.

**Table 1:** Characteristics of the included studies

Source	Country	Population	Sample size	Interventions		Vital signs measured by WMS					Age (years)		Sex (% male)	
			Total	Control (EWS)	WMS	HR	RR	SpO2	BP	T	Control	WMS	Control	WMS
*Included studies and outcomes*														
***Meta-analysis***														
Skraastad et al. 2019	Norway	General surgery	195	Intermittent vital signs (NEWS)	PSE (NEWS) + ESS	x	x	x	x	x	62	61	63%	63%
Downey et al. 2020	UK	General surgery	135	Intermittent vital signs (NEWS)	SensiumVitals + NEWS	x	x			x	62	65	54%	52%
Downey et al. 2018	UK	General surgery	226	Intermittent vital signs (NEWS)	SensiumVitals + NEWS	x	x			x	63.7	65.2	45%	54%
Kisner et al. 2009	Switzerland	General surgery	357	Intermittent vital signs (Unknown)	Auricall	x		x			62.7	65	75%	75%
Weller et al. 2018	USA	Neurology and neurosurgery	1958	Intermittent vital signs (Unknown)	VisiMobile	x	x	x	x	x	59.3	60.5	58%	54%
Verrillo et al. 2018	USA	Orthopaedic, spinal and trauma general care	849	Intermittent vital signs (Unknown)	VisiMobile	x	x	x	x	x	51.4	54.5	58%	54%
Watkinson et al. 2020	UK	Gastro intestinal surgery	407	Intermittent vital signs (CEWS)	Avant-4100	x		x			63	63	56%	58%
***Narrative***														
Weenk et al. 2019	Netherlands	Surgical and internal medicine ward	60*	Intermittent measurement (MEWS)	VisiMobile	x	x	x	x	x	N/A	63	N/A	60%
					HealthPatch	x	x			x	N/A	56	N/A	73.3%
Weenk et al. 2020	Netherlands	Surgical and internal medicine ward	90*	Intermittent measurement (MEWS)	VisiMobile	x	x	x	x	x	62	63	67%	60%
					HealthPatch	x	x			x		56		73.3%
Monson et al. 2020	USA	Labour and delivery ward	216	Standard external monitoring	Monica Novii	x**					28.6	29.2	0%	0%

WMS: wearable monitoring system, BP: blood pressure, CEWS: centile-based early warning score, ESS: efficacy safety score, EWS: early warning score, HR: heart rate, NEWS: national early warning score, PSE: Patient Status Engine, RR: respiratory rate, SpO2: peripheral oxygen saturation, T: temperature, x: device measures that vital sign. *Same patients. Weenk et al. 2020 added 30 controls. ** Both maternal and fetal heart rate

**Table 2:** Included studies primary outcomes and outcomes included in the meta-analysis

Source	Design	Primary outcome	Deterioration detection outcomes				Clinical outcomes	
			ICU transfer	RRT activation or cardiac arrest team call	All complications	Major complications	Mortality	Length of stay
**Meta-analysis**								
Skraastad et al. 2019	RCT	Time to mobilisation			X	X	X	X
Downey et al. 2020	Pilot RCT	Progression criteria to full RCT	X		X	X	X	X
Downey et al. 2018	Cluster RCT	Time to antibiotics in patients with sepsis	X		X	X	X	X
Kisner et al. 2009	Before-after	Incidence of postoperative atrial fibrillation			X			
Weller et al. 2018	Before-after	Rate of patient deterioration events	X	X			X	X
Verrillo et al. 2018	Before-after	Early deterioration detection	X	X	X		X	
Watkinson et al. 2020	Before-after	Length of stay	X	X			X	X
**Narrative**								
Weenk et al. 2019	RCT	Experiences of patients and care givers						
Weenk et al. 2020								
Monson et al. 2020	RCT	Amount of time with the interpretable fetal HR tracing during of labour						

ICU: Intensive Care unit, RCT: Randomised Controlled Trial, RRT: Rapid Response Team, x: study included that outcome

The majority of the included studies implemented the WMS in post-surgical patients. Four studies also reported the patient American Society of Anaesthesiologists (ASA) score for preoperative functional status [45], with a median ASA score of 2 (“Patient has mild systemic disease”) in three studies [32, 33, 46] and 3 (“Patient has severe systemic disease that is not incapacitating.”) in one [30].

Reviewers achieved a fair level of agreement (kappa: 0.348; 95% CI 0.285 to 0.482) for study inclusion; all abstracts with disagreements were included for full-text assessment. There were no major disagreements between reviewers regarding data extraction, study quality or bias assessments.

Studies not included in the meta-analysis were narratively explored (Tables 1 and 2). Two papers reported results from one RCT, comparing two devices (HealthPatch and VisiMobile) with nurse measurements [40, 41]. However, they did not include the third group (control) in the analysis and did not assess any clinical outcomes, mostly exploring factors related to deterioration detection, failing to provide sufficient data to include in the meta-analysis. In the first paper from this RCT, the authors report that both HealthPatch and VisiMobile modified early warning scores (MEWS) were higher than the nurse measured MEWS, mostly due to RR measurement differences [41]. In the second paper (the full RCT) the authors identified positive and negative effects as well as barriers and facilitators for the use of these devices, such as the impact of WMS on a shorter length of stay and prevention of ICU admissions. Additionally, a total of 17 patients, 2 relatives and 17 healthcare professionals reported that they expected earlier deterioration detection using these wearables [40].

Another RCT evaluated wireless external fetal electrocardiography versus standard external monitoring [44]. We were unable to include this study in the meta-analysis as (1) the primary outcome of the study was the percentage of interpretable fetal HR data, (2) the population of interest is very different from the remaining studies and (3) the clinical outcomes analysed also differed (e.g. length of labour, fetal Apgar score, etc.). Considering this, their results demonstrated no differences in maternal or neonatal clinical outcomes between groups. However, results did suggest an increased acceptance by patients and staff, with satisfaction scores significantly higher when compared to the standard monitor [44].

#### Included studies registration

Details of the clinical trials search are shown in Additional file 6: Appendix 6. Of the ten included studies in this review, only seven were registered (most retrospectively, as per Additional file 7: Appendix 7). Within these, all primary outcomes stated in the registration were reported in the main paper, as well as most of the secondary outcomes.

### Study quality and risk of bias

**Fig. 2:**
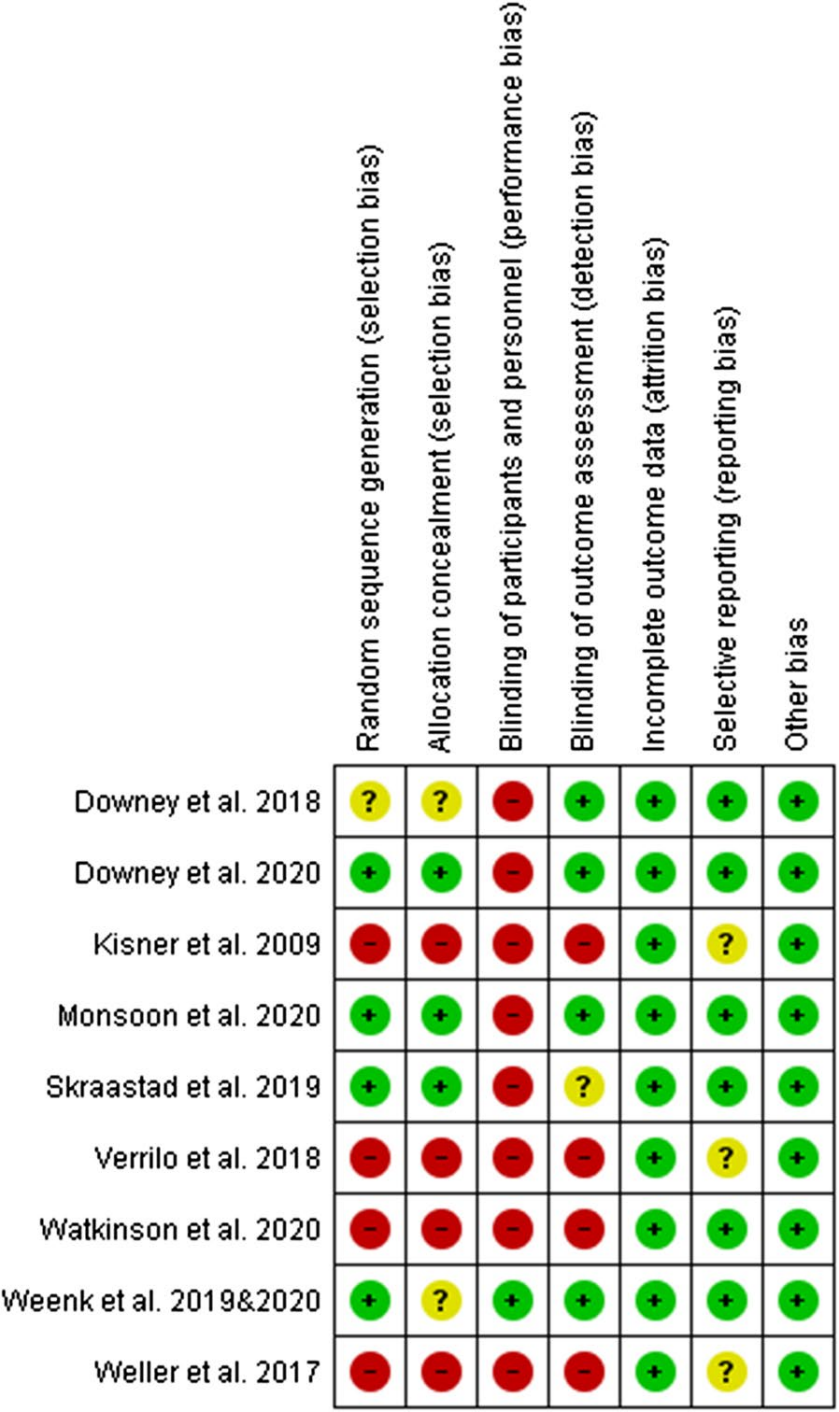
Risk of bias summary: review authors' judgements about each risk of bias item for each included study. Green ( −): Low risk of bias, Red ( −): High risk of bias, Yellow (?): unclear risk of bias

**Fig. 3:**
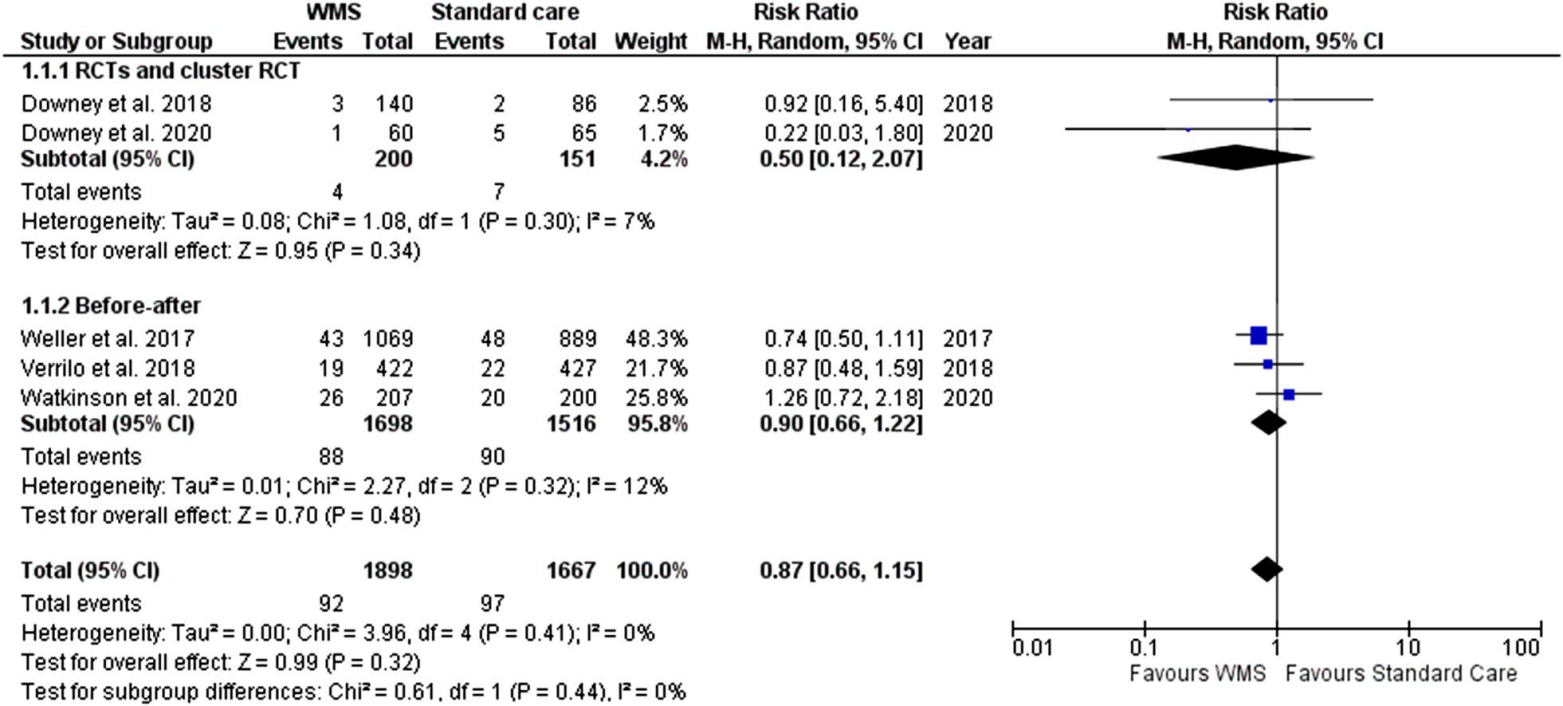
Meta-analysis forest plots comparing WMS and standard care ICU transfer risk ratio. WMS: wearable monitoring systems, CI: confidence intervals, M-H: Mantel–Haenszel, RCT: randomised controlled trial

The overall quality of included studies was moderate with some bias to take into account, as per Figs. 2 And 3. For the included RCTs, using the ROB2, two were identified as being at “low risk” of bias [33, 44] and a further three were assessed as raising “some concerns” [30, 40], including the cluster RCT [32]. The risk of bias, assessed by the ROBINS-I was “moderate” for all before-and-after studies [29, 39, 42, 43]. See Additional file 8: Appendix 8 for further details. The results of the bias assessment did not influence inclusion in the meta-analyses.

### Primary outcomes

In total, data from seven studies were included in the meta-analysis of primary outcomes related to deterioration detection, analysed separately according to the three reported deterioration outcomes – ICU transfers, rapid response or cardiac arrest activation, and complications.

#### ICU transfers

A total of five studies reported ICU transfers and were included in this meta-analysis (data from 3565 patients, 1898 in the WMS group) [29, 32, 33, 39, 43]. Pooling of data indicated that use of WMS did reduce ICU transfer (RR 0.87; 95% CI 0.66–1.15), but not statistically significant (*p* = 0.32) (Fig. 3).

#### Rapid response or cardiac arrest activation

**Fig. 4:**
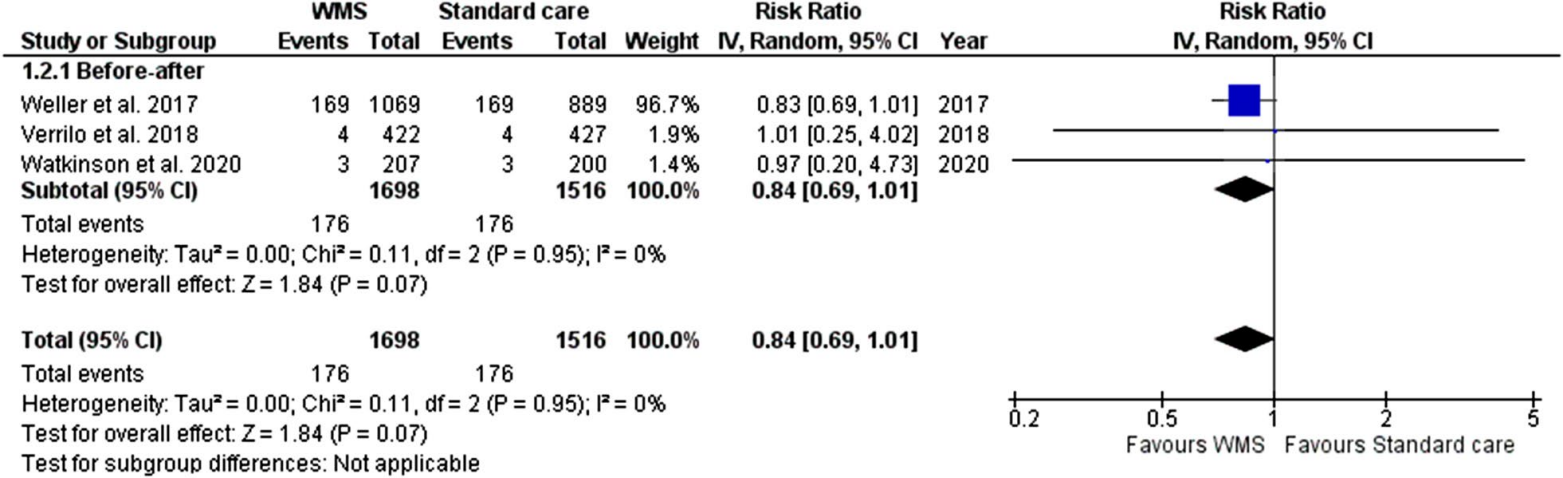
Meta-analysis forest plots comparing WMS and standard care rapid response or cardiac arrest team activation risk ratio. WMS: wearable monitoring systems, CI: confidence intervals, M-H: Mantel–Haenszel

For this outcome, two before-and-after studies reporting rapid response team activation and another study reporting cardiac arrest calls were included (with data from 3214 patients, 1698 in the WMS group, Fig. 4) [29, 39, 43]. Pooled data for this outcome indicated WMS reduced RRT or cardiac arrest calls (RR 0.84; 95% CI 0.69–1.01) with a p-value near statistical significance (*p* = 0.07).

#### All clinical complications

**Fig. 5:**
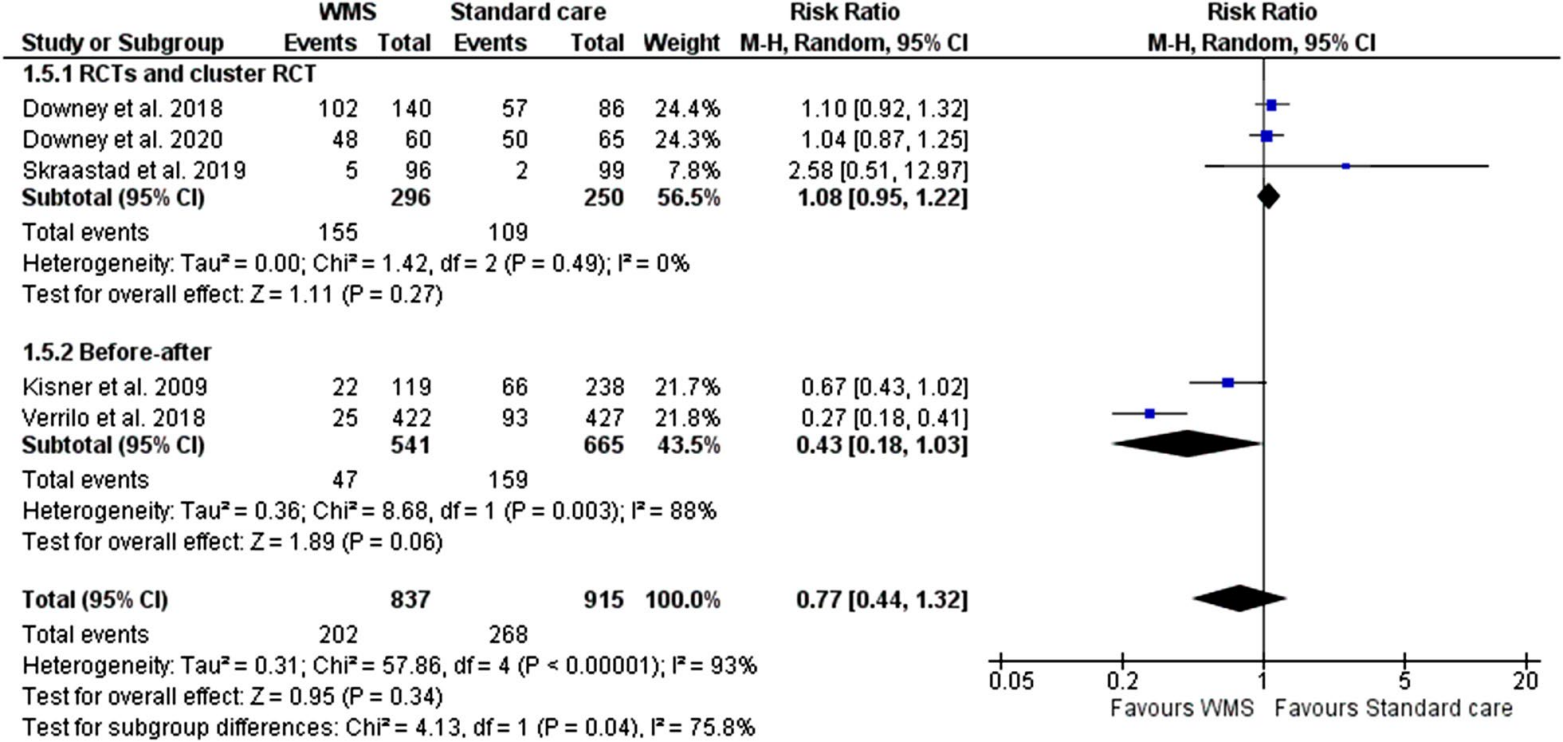
Meta-analysis forest plots comparing WMS and standard care all complications risk ratio. WMS: wearable monitoring systems, CI: confidence intervals, M-H: Mantel–Haenszel, RCT: randomised controlled trial

A total of five studies reported data on complication outcomes classed by the Clavien-Dindo system as grade I or II(with data from 1752 patients, 837 in the WMS group, Fig. 5). indicating the WMS group had a reduced risk of complications (RR, 0.77; 95% CI 0.44 to 1.32) however without statistical significance (p = 0.34) and with high heterogeneity between studies (I^2^ = 93%).

**Fig. 6:**
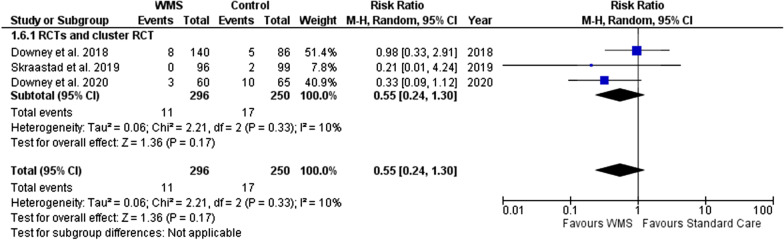
Meta-analysis forest plots comparing WMS and standard care major complications risk ratio. WMS: wearable monitoring systems, CI: confidence intervals, M-H: Mantel–Haenszel, RCT: randomised controlled trial

For the major complications (Fig. 6), we included 3 studies (with data from 546 patients, 296 in the WMS group) indicating the WMS group had reduced risk of major complications (RR, 0.55; 95% CI 0.24 to 1.30) however, with no statistical significance (p = 0.17).

#### Other deterioration detection outcomes not included in the meta-analysis

A few of the included studies also explored other deterioration detection outcomes, but in insufficient numbers to allow a meta-analysis. One cluster RCT [32] and one RCT [33] from the same research group compared the time to antibiotic administration in case of sepsis in the WMS group against the control group, finding this statistically insignificant in both studies (656.0 (95% CI 431.7–820.3) vs 1012.8 (95% CI 425.0–1600.6) minutes [32] and 551 (95% CI 296–805) vs 527 (95% CI 199–856)) [33].

### Secondary outcomes

The two secondary outcomes of in-hospital mortality and hospital length of stay were also meta-analysed.

#### In-hospital mortality

**Fig. 7:**
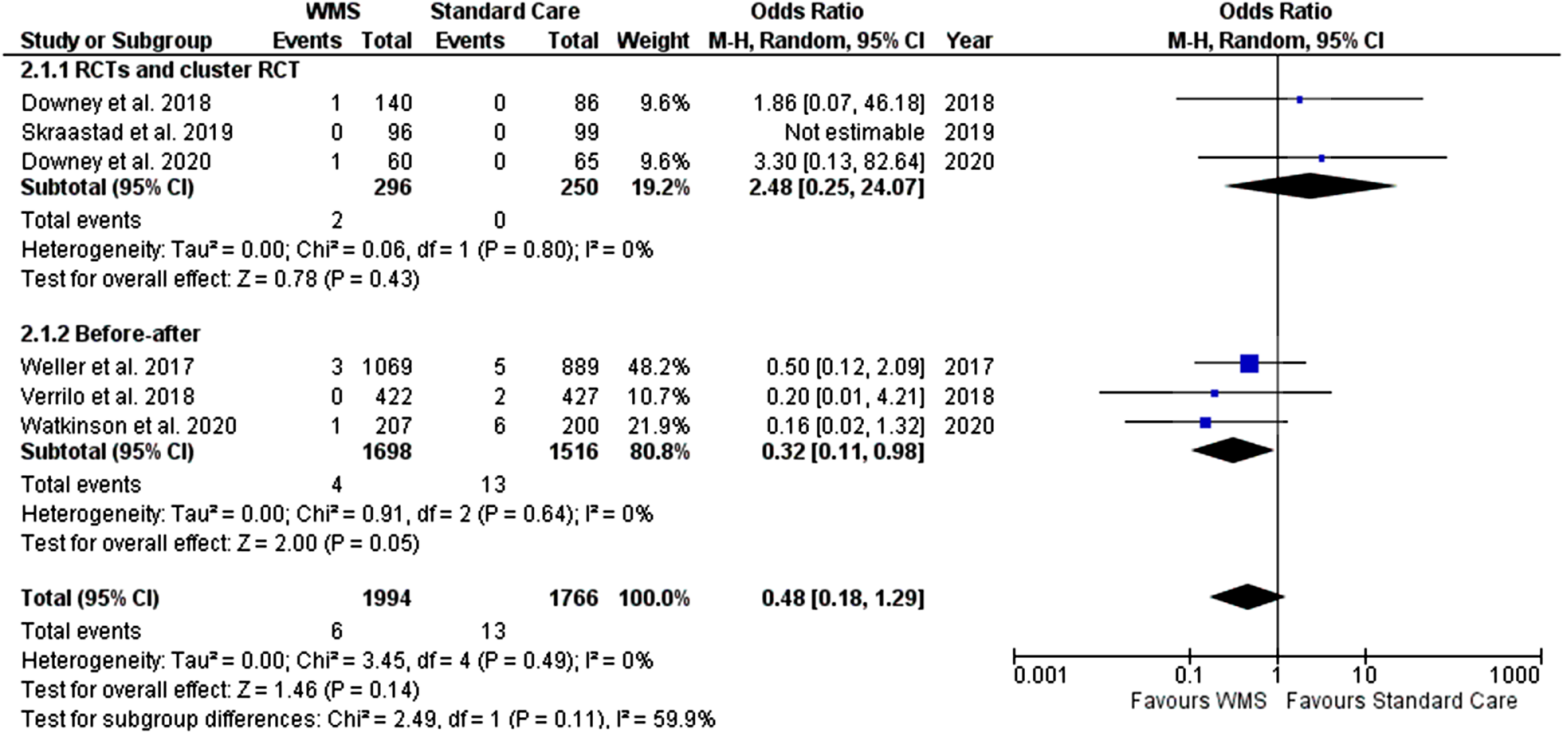
Meta-analysis forest plots comparing WMS and standard care in-hospital mortality risk ratio. WMS: wearable monitoring systems, CI: confidence intervals, M-H: Mantel–Haenszel, RCT: randomised controlled trial

For the outcome of in-hospital mortality, we included six studies (with data from 3760 patients, 1994 in the WMS group, Fig. 7) [29, 30, 32, 33, 39, 43], with one study reporting no deaths in either group (no estimates to be analysed in the meta-analysis) [30]. Our results indicated the WMS group had a reduced risk of mortality (RR, 0.48; 95% CI 0.18 to 1.29) but this reduction was not statistically significant (p = 0.15).

#### Hospital length of stay

**Fig. 8:**
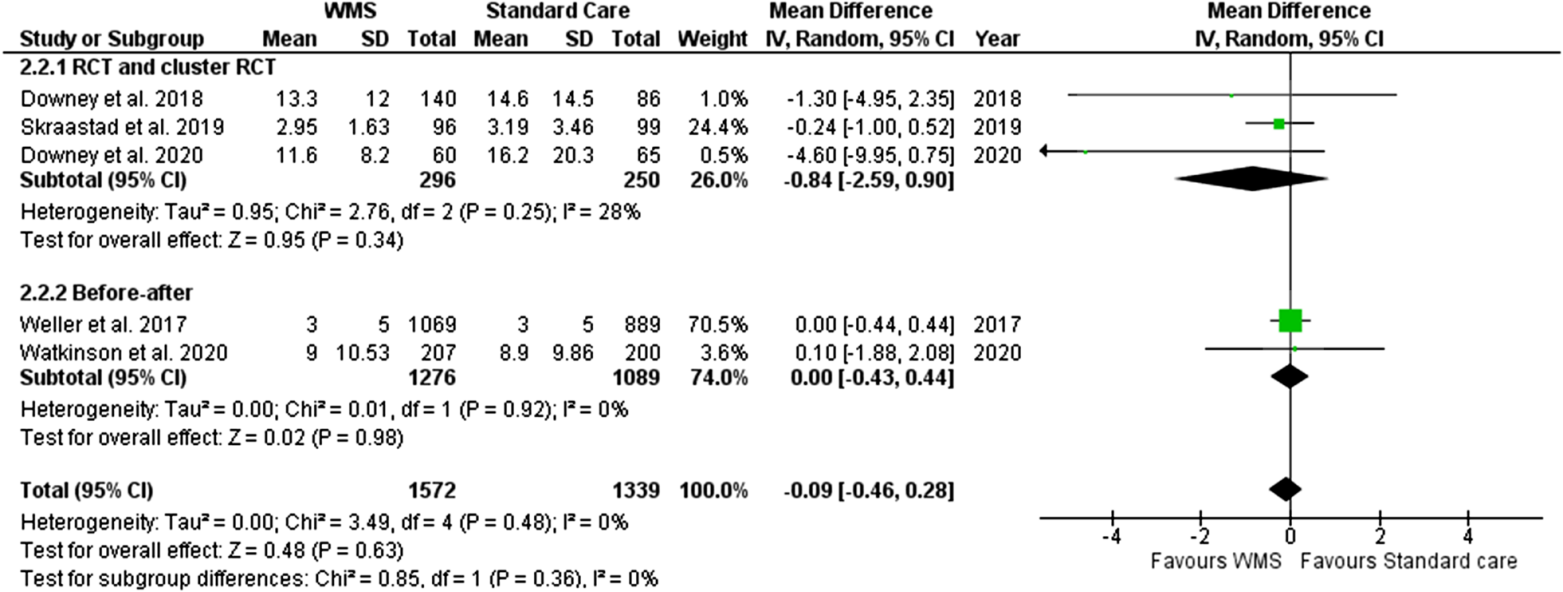
Meta-analysis forest plots comparing WMS and standard care hospital length of stay mean difference. WMS: wearable monitoring systems, CI: confidence intervals, IV: inverse variance, RCT: randomised controlled trial

A total of five studies were included for the outcome of hospital LoS (with data from 2911 patients, 1994 in the WMS group, Fig. 8) indicating a non-significant reduction in hospital length of stay for patients monitored using WMS (MD − 0.09; 95% CI − 0.46 to 0.28, *p* = 0.63).

#### Other clinical outcomes not included in the meta-analysis

Studies also included other clinical outcomes; for example, two studies explored 30-day hospital readmission rates and showed mixed results, with one showing lower readmission rates in the WMS group [32] and the other slightly higher [33] in comparison with standard care.

Skraastad and colleagues indicated reduced time to post-operative mobilisation in the WMS group, 10.1 (95% CI 8.1–12.2) against 14.2 (95% CI 12.0–16.3) in the control group [30]. They also compared the number of NEWS measurements in their RCT, with 8.2 (95% CI 47.4–9.0) in the WMS group versus 3.4 (95% CI 3.1–3.6) in the standard care group. Additionally, there was a higher mean in opioid dose given in the WMS group, 25.5 (95% CI 20.9–30.0) vs 15.2 (95% CI 11.1–19.3) in the control arm; and more supplementary oxygen was given to 57/96 in the WMS group against 32/99 in the control group [30]. The authors justify this as being a result of the increased monitoring in the WMS group, facilitating pain and oxygen management of those patients, and promoting earlier mobilisation [30].

### Exploratory outcomes

#### Alerting systems (Central station and mobile devices for alarms)

Some information about the alerting system was available in nine out of the ten included studies [29, 30, 32, 33, 39, 41–43, 47]. Five studies reported their development in reducing the number of alarms per patient per day (APDs). One reported having started with 11.41 APDs and decreased this iteratively down to 2.01 APDs, reducing the non-actionable alarms and modifying vital-sign limits and thresholds to reduce the rate of false alarms [29]. The authors focused on monitoring optimisation, reviewing and modelling the alarm data every few days and discussing with clinical managers whether widening vital sign parameters would create a significant reduction in alarm rates while still being clinically acceptable and useful for deterioration detection [29]. In Downey and colleagues’ first study there was an unacceptable number of alarms sent to the nursing staff. After adjustments in the vital-sign thresholds, the number of false alarms was reduced by 90% (30). The same issue was addressed in their second RCT, in which a clinical fellow visited the wards daily to check the rate of false alarms and adjust thresholds and/or delays of the alarms according to clinical need (18, 48). Despite this, two patients withdrew from the study due to “too many false alarms” (18). In two other studies, the authors just discussed their intention of improving the rate of true positives and reducing the rate of false negatives/false alarms [40, 41].

In most studies, alerting thresholds were pre-set and individualised as required, with alerting through the central station and/or nurse mobile/pager/PDA, using audio alarms in the majority of cases (Table 3). In one study the authors used a single risk score calculated from all vital signs (VSI), based on modelling from a previous patient dataset, generating an alarm when the VSI score was above the threshold for more than 4 out of 5 min [43]. Alerting parameters from the included studies are explored in Table 3.

**Table 3:** Vital sign thresholds summary table

	Vital sign thresholds (delay before alarm in seconds)									Alarm method
Study	HR/PR		RR		SpO2	BP		MAP	T	
	High	Low	High	Low	Low	High SBP	Low DBP	Low		
Weller et al. 2018	HR: 150 (15) PR: 150 (60)	HR: 39 (15) PR: 39 (60)	35 (120)	4 (120)	85 (90)	200 (240)		58 (60)		Sound Central station + nurse phone
Kisner et al. 2009	Individualised (immediate)				Pre-set individualised (immediate)					Sound Pager or SMS to doctor and nurse
Verrilo et al. 2018	Pre-set (5)		Pre-set (120)		Pre-set (60)	Pre-set (120)				Sound Central monitor + nurse mobile
Downey et al. 2018	Pre-set, individualised if required (immediate)		Pre-set, individualised if required (immediate)						Pre-set, individualised if required (immediate)	Sound Mobile device to nurse
Downey et al. 2020	Pre-set, individualised if required (immediate)		Pre-set, individualised if required (immediate)						Pre-set, individualised if required (immediate)	Sound Mobile device to nurse
Weenk et al. 2019 & 2020	Individualised (immediate)		Individualised (immediate)		Individualised (immediate)	Individualised (immediate)			Individualised (immediate)	Sound Nurse station
Skraastad et al. 2020	As per NEWS score		As per NEWS score		As per NEWS score	As per NEWS score			As per NEWS score	Visual only Warnings at patient bedside

	Score name	Alerting score thresholds							
		Vital signs included in score	Range	Threshold	Time to alarm				
Watkinson et al. 2020	VSI	All	0 to 5	> 3.0 for more than 4 out of 5 min	On the fifth minute				Sound Bedside + central station + nurse PDA

BP: blood pressure, DBP: diastolic blood pressure, HR: heart rate, MAP: mean arterial pressure, PDA: personal digital assistant, PR: pulse rate, RR: respiratory rate, SBP: systolic blood pressure, SMS: short message service, SpO2: peripheral oxygen saturation, T: temperature, VSI: Visensia Safety Index

In one study’s final version of the alerting system, hypotension, bradycardia and hypoxaemia were tolerated for shorter periods than tachycardia or hypertension, unless the tachycardia resulted in hypotension. Additionally, the majority of the alarms in the final iteration were due to low SpO_2_ (97% of APDs) [29]. Another study found that the most accurate vital sign parameter was systolic blood pressure, which had a positive predictive value (PPV) of 97%, followed by high respiratory rate (PPV of 85%) and low SpO_2_ (PPV of 76%), indicating high sensitivity and reliability and a low false alarm rate [39].

#### Clinical trial registries (other potentially eligible studies to be included)

A total of sixteen registrations were identified in the search and screened for eligibility. Six were excluded and six registrations refer to the included studies. A further four registrations were deemed potentially eligible to be included in our review and meta-analysis (Table 4). A registered cluster RCT [48] aimed to develop a two-tiered monitoring system to improve the care of patients at risk of clinical deterioration in general hospital wards. This registered study also included a subset of patients using wireless devices [48]. However, although the main results are published [35], no data were reported on the impact of wireless devices on the subset population and we were unable to make contact with the Principal Investigator to clarify publication status and request this subset of the WMS group data. A further registration [49] before-and-after study was potentially eligible and although the main results are published [36] a subset of patients (278) used at least one cableless sensor, but the author confirmed there were no data available on outcomes for this sub-set of wirelessly monitored patients [36]. The other two registrations did not publish their results at the time of our systematic literature search. The Principal Investigator of one prospective, observational cohort study confirmed the study results have been submitted and are under peer review [50]. We were unable to contact the Principal Investigator to clarify the status of the other study [51].

**Table 4:** Studies with potential for inclusion

Year registered	Registration ID	Date start recruitment	Recruitment status	Reason for non-publication
2011	NCT01280942 [48]	January 2011	Completed	Main results published but not sub-group analysis with the wireless device [35]
2012	NCT01692847 [49]	October 2012	Completed	Main results published but not sub-group analysis with the cableless device [36]
2015	NCT02427828 [51]	March 2013	Completed	Unknown
2017	NCT03179267 [50]	September 2017	Completed	Under peer review

## Discussion

### Main results

In this systematic review and meta-analysis, we identified 10 eligible studies of various designs comparing the impact of WMS on deterioration detection and clinical outcomes with standard care, including a total of 4433 patients. Our main findings suggest that there is currently no strong evidence to suggest WMS is superior to standard care; there is some indication of a trend towards WMS enabling a reduction in ICU admissions, RRT/cardiac arrest calls and complications in hospitalised patients, however without statistical significance. Our results are limited by the small number of studies, limited sample sizes, and overall moderate risk of bias, failing to provide a generalisable answer to our research question (GradePro summary in Additional file 5: Appendix 5). This review also suggests a trend towards WMS decreasing in-hospital mortality and length of stay, but again without strong statistical significance supporting these findings.

Although our review focused specifically on wearable monitoring devices, our results are in accordance with a previous systematic review which had a focus on the clinical impact of a broader range of multi-parameter continuous non-invasive monitoring of vital signs in non-intensive care unit patients. In this review, the authors included all non-invasive devices (including wired and static bed monitors such as EarlySense) and also found a trend towards decreased ICU transfers, RRT activations and hospital length of stay, with a suggestion of reduced hospital mortality for patients monitored with these devices [14]. Our study also updates this review, with the inclusion of an additional four more recent studies.

All the studies included in our review were conducted in a non-ICU environment (mostly in surgical patients) and most comparators were standard intermittent vital-sign monitoring with the use of local EWS. Previous evidence suggests that focus on non-critical care settings is due to WMS being unable to replace the continuous monitoring commonly used for high-dependency patients. Instead, WMS offers an intermediate level of monitoring between continuous high-dependency monitoring and intermittent manual measurements, with the potential to facilitate early deterioration detection in high-risk patients (e.g. post-ICU) [5]. In addition, a recent study in the paediatric population concluded that wireless monitoring is feasible and can identify more deteriorations. The authors suggest that by using this in combination with a paediatrics early warning (PEW) score, some life-threatening events may be prevented [38].

One study included in our review reported patients’, relatives’ and healthcare professionals’ perceptions of the use of WMS on the general ward, and found agreement between all interviewed groups that WMS could facilitate earlier deterioration detection and improve patient safety without posing a barrier to mobility, as well as reduce staff workload and hospital costs [40], agreeing with previous evidence [52–54] and reinforcing the direction of our findings.

To better understand the WMS used in the included studies, we aggregated available information on the alerting methods and thresholds. As most studies used audio alarms, system iterations seemed to focus on reducing the rate of false alarms by adjusting/individualising each vital sign and/or overall score to avoid alarm fatigue for clinical staff. This has been previously discussed as an important factor for the successful deployment of monitoring technology [55–58]. The exception was one study that used visual warnings alone, resulting in increased NEWS measurements in patients using WMS without clinical staff being aware of the potential deterioration. They identified three patients for whom WMS alarmed between two intermittent measurements and who were later diagnosed with pneumonia, atrial fibrillation and an anastomotic leakage [41]; in the same study, the authors also explored the delay between high MEWS measured by a device and next regular MEWS measurement by a nurse, ranging from 0 up to 10 h, and varying between day and night [41].

Finally, a review of known registries was conducted to address the issue of publication bias, finding that the majority of the included studies were registered either in ClinicalTrials.gov or ISRCTN databases and all registered primary outcomes were reported as well as most of the secondary (Additional file 7: Appendix 7). However, most studies were registered retrospectively, rather than prospectively. We did, however, identify four studies that might have contributed to this systematic review by either performing a subgroup analysis for patients using wearable devices [36, 48] and by publishing their results as registered in another two [50, 51]. Our registry search allowed us to highlight this under-reported and non-published evidence, which could have potentially impacted our results and contributed to the meta-analysis and overall body of evidence available in this field.

### Study limitations

There were some limitations to this review. The number of studies included was limited and used a variety of designs, populations, outcomes, medical devices/systems, EWS and alerting thresholds. Our meta-analysis only included two RCTs (one being a pilot) and one pilot cluster RCT, all of which had small sample sizes which reduced the probability of significant difference in outcomes, despite large effect sizes. In contrast, the included before-and-after studies had larger sample sizes but increased bias and quality limitations, again posing a barrier to any significant conclusion. Additionally, most included studies had “some concerns” or “moderate” risk of bias, also affecting the quality of our results. This reflects the emerging nature of this area of research and highlights the need for a large, multicentre RCT that evaluates whether WMS may be beneficial for early deterioration detection and related clinical outcomes in hospitalised patients.

Despite the apparent variation in the interventions and methodologies between the different studies, the meta-analyses generally showed low heterogeneity of outcomes (assessed using the I^2^ statistic). This could be because the results were generally quite close to the null for most outcomes, and therefore little variability would be expected. Additionally, the small number of studies will have hampered the ability to robustly assess heterogeneity.

One of the studies [32] had an extra non-randomised bay in their exploratory analysis. We did not include these data in our meta-analysis in accordance with Cochrane guidelines, so only the randomised groups were considered [59]. Similarly, another study compared the WMS group with a period before in the same unit and a period during and before in an alternative unit. In this case, we only used the data comparing the before period in the same unit, to minimise selection bias [59]. For RRT and cardiac team call analysis, it is important to note that the Weller and colleagues study accounted for 96.7% of the weight on these results [29]. In addition, studies exploring RRT and cardiac team calls were combined in the meta-analysis, and therefore results might differ if analysed separately.

As previously reported, bias may be present as clinical staff are aware of the WMS use in their patients. However, the practicalities of blinding in WMS studies may not be feasible and may potentially have a counterproductive impact on clinical outcomes [14]. Additionally, all studies failed to provide practical information of other potential confounders for the reported primary outcomes, for example, staff seniority throughout patient stay, local ICU capacity (that might impact transfers), the Do Not Attempt Resuscitation (DNAR) status of the patients who died, among other factors. This also reflects the need for a human-factors approach when designing and evaluating WMS implementation studies.

### Future research

The results of this systematic review highlighted the need for more, bigger and better studies to support WMS implementation, and test its impact on early deterioration detection and clinical outcomes. Our findings are limited by the reduced sample size of the included RCTs and reduced methodological quality of the before-and-after studies. One of the included studies [33] conducted a subgroup analysis exploring clinical outcomes in high-risk participants. This further highlighted the potential impact of the WMS use in patients at higher risk of deterioration and further RCTs should consider including a similar analysis of their results.

In this review, we also explored the methods for alerting and the alarm parameters of the included studies; our results suggest that “less is more” and studies seemed to focus on reducing false alarms and, consequently, increase the number of actionable alarms. Future implementation research should consider this, as well as the time required for the alarm system optimisation before WMS deployment.

Remote monitoring systems are only beneficial to the clinical staff if they are easy to use and clinicians understand the potential benefit on clinical outcomes [60]. This review is part of a wider phased project in our research programme, the virtual High Dependency Unit (vHDU) study. So far, we have selected [61] and tested a number of wearable devices [62, 63], prior to selection and integration in a final WMS, which will be evaluated in a pilot study and then a full multicentre RCT.

## Conclusions

Our systematic review indicates that there is no current statistically significant evidence that implementation of WMS impacts early deterioration detection and associated clinical outcomes. This review highlights the need for bigger and more rigorous RCTs to support WMS implementation and deployment. Additionally, our narrative findings suggest that alarm thresholds should be adjusted to minimise false alarms and thus enable prompt clinical response to true deterioration events.

## Supplementary Information

**Additional file 1**. PRISMA checklists

**Additional file 2**. Final Search strategies—studies

**Additional file 3**. Final Search strategies—registries

**Additional file 4**. Final Search strategies—grey literature

**Additional file 5**. GradePro tables

**Additional file 6**. Clinical Trial registry flowchart

**Additional file 7**. Registered studies information

**Additional file 8**. Quality assessment scales

## Data Availability

All data generated or analysed during this study are included in this published article and its supplementary information files.

## Abbreviations

ADLs: Activities of daily living
APD: Alarms per patient per day
ASA: American Society of Anaesthesiologists
CEWS: Centile-based early warning score
BP: Blood pressure
ci: Confidence interval
CRS: Comfort rating scale
ECG: Electrocardiogram
ews: Early warning score
HR: Heart rate
ICJME: International committee of medical journal editors
ICU: Intensive care unit
IQR: Interquartile range
LoS: Length of stay
MD: Mean difference
MEWS: Modified early warning score
NEWS: National early warning score
NIHR: National Institute for Health Research
NOS: Newcastle Ottawa scale
PEW: Pediatric early warning
PPV: Positive predictive value PR: pulse rate
RCT: Randomised Controlled Trial
RoB2: Cochrane risk of bias tool
ROBINS-I: Risk Of Bias In Non-randomised Studies—of Interventions
RR: Respiratory rate
RR: Risk ratio
RRT: Rapid response team
SBP: Systolic blood pressure
SpO_2_: Peripheral capillary oxygen saturation
UK: United Kingdom
vHDU: Virtual High Dependency Unit
WMS: Wearable monitoring system
