# Supplementary material for: The impact of wearable continuous vital sign monitoring on deterioration detection and clinical outcomes in hospitalised patients: a systematic review and meta-analysis
Source: Crit Care. 2021 Sep 28;25:351. doi: 10.1186/s13054-021-03766-4 (PMC8477465; doi:10.1186/s13054-021-03766-4)
Supplement: Supplementary file 2 — Additional file 2. Final Search strategies—studies [file 13054_2021_3766_MOESM2_ESM.docx]

# Appendix 2 – Final Search strategies – studies

Database: Medline (Ovid MEDLINE® Epub Ahead of Print, In-Process & Other Non-Indexed Citations, Ovid MEDLINE® Daily and Ovid MEDLINE®) 1946 to present

Search Strategy: (27/08/20)

--------------------------------------------------------------------------------

1 exp Monitoring, Ambulatory/ (28543)

2 ambulatory.tw. (78329)

3 exp Wearable Electronic Devices/ (11822)

4 wearable.tw. (12014)

5 (body adj3 borne).tw. (72)

6 (head mount* adj3 (display* or device* or system*)).tw. (1101)

7 (head-up adj3 (display* or device* or system*)).tw. (153)

8 (head-worn adj3 (display* or device* or system*)).tw. (31)

9 (patch or patches).tw. (106471)

10 Adhesives/ (6682)

11 adhesive*.tw. (57587)

12 (sticker* or stick or sticks).tw. (12250)

13 (watch or watches).tw. (9028)

14 smartwatch*.tw. (309)

15 telemonitor*.tw. (1516)

16 tele monitor*.tw. (146)

17 exp Telemetry/ (13327)

18 telemet*.tw. (9378)

19 clothing/ or shoes/ (15121)

20 cloth*.tw. (16130)

21 exp Textiles/ (6392)

22 textile*.tw. (10792)

23 exp Telemedicine/ (29383)

24 telemedicine*.tw. (10894)

25 exp Biosensing Techniques/ (54829)

26 biosens*.tw. (37047)

27 glove*.tw. (10632)

28 shoe*.tw. (9295)

29 accessor*.tw. (44268)

30 Wrist/ (9011)

31 wrist*.tw. (38559)

32 necklace*.tw. (904)

33 (belt or belts).tw. (12916)

34 armband*.tw. (590)

35 (shirt or shirts).tw. (1067)

36 contactless.tw. (1731)

37 cableless.tw. (8)

38 contact sensor*.tw. (184)

39 unobtrusive.tw. (1596)

40 remote.tw. (69973)

41 Wireless Technology/ (3587)

42 wireless*.tw. (14654)

43 non contact.tw. (5281)

44 noncontact.tw. (4723)

45 without wires.tw. (21)

46 non-restrict*.tw. (555)

47 unrestrict*.tw. (8566)

48 Automation/ (18179)

49 automat*.tw. (229850)

50 webcam*.tw. (397)

51 web-cam*.tw. (175)

52 video*.tw. (126640)

53 2 or 3 or 4 or 5 or 6 or 7 or 8 or 9 or 10 or 11 or 12 or 13 or 14 or 15 or 16 or 17 or 18 or 19 or 20 or 21 or 22 or 23 or 24 or 25 or 26 or 27 or 28 or 29 or 30 or 31 or 32 or 33 or 34 or 35 or 36 or 37 or 38 or 39 or 40 or 41 or 42 or 43 or 44 or 45 or 46 or 47 or 48 or 49 or 50 or 51 or 52 (979503)

54 Monitoring, Physiologic/mt [Methods] (18136)

55 monitor*.tw. (804541)

56 Oximetry/ (13024)

57 oximet*.tw. (13397)

58 tracker*.tw. (4568)

59 Radar/ (1203)

60 radar*.tw. (5333)

61 Ballistocardiography/ (2049)

62 ballisto*.tw. (1831)

63 exp Accelerometry/ (8929)

64 accelerom*.tw. (16743)

65 gyro*.tw. (4773)

66 Photoplethysmography/ (1987)

67 ppg.tw. (3458)

68 photople*.tw. (3299)

69 videopleth*.tw. (4)

70 video pleth*.tw. (6)

71 (photo* adj3 pleth*).tw. (339)

72 Thermography/ (7571)

73 thermograph*.tw. (6133)

74 Infrared Rays/ (13281)

75 infrared*.tw. (137632)

76 thermal.tw. (200146)

77 thermistor*.tw. (1748)

78 thermoresister*.tw. (0)

79 camera*.tw. (45491)

80 wavelet analysis/ (2189)

81 wavelet*.tw. (11286)

82 waveform*.tw. (28510)

83 ambient light*.tw. (1886)

84 piezoelectric*.tw. (10492)

85 piezo electric*.tw. (342)

86 Electric Impedance/ (17145)

87 impedan*.tw. (45070)

88 exp Electrocardiography/ (204339)

89 electrocar*.tw. (88334)

90 (ecg or ecgs).tw. (65572)

91 (ekg or ekgs).tw. (3081)

92 electrom*.tw. (97510)

93 electrog*.tw. (20290)

94 exp Electrodes/ (130099)

95 electrode*.tw. (161247)

96 SensiumVitals.tw. (8)

97 IntelliVue Guardian.tw. (0)

98 VitalPatch.tw. (2)

99 Vitalsolutions.tw. (0)

100 Guardian Angel.tw. (74)

101 Visi Mobile.tw. (6)

102 Current health.tw. (4290)

103 Snap40.tw. (1)

104 Multi-vital ECG patch.tw. (0)

105 Caretaker.tw. (1456)

106 Life scope G3.tw. (0)

107 Biostamp.tw. (4)

108 TAGECG.tw. (0)

109 ZioXT patch.tw. (0)

110 ZioAT patch.tw. (0)

111 Cardea solo.tw. (0)

112 Peerbridge Cor.tw. (0)

113 Radius-7.tw. (24)

114 Radius-PPG.tw. (0)

115 WristOx2.tw. (4)

116 Lifesync.tw. (1)

117 Ariatele.tw. (0)

118 54 or 55 or 56 or 57 or 58 or 59 or 60 or 61 or 62 or 63 or 64 or 65 or 66 or 67 or 68 or 69 or 70 or 71 or 72 or 73 or 74 or 75 or 76 or 77 or 78 or 79 or 80 or 81 or 82 or 83 or 84 or 85 or 86 or 87 or 88 or 89 or 90 or 91 or 92 or 93 or 94 or 95 or 96 or 97 or 98 or 99 or 100 or 101 or 102 or 103 or 104 or 105 or 106 or 107 or 108 or 109 or 110 or 111 or 112 or 113 or 114 or 115 or 116 or 117 (1802638)

119 53 and 118 (177463)

120 1 or 119 (194037)

121 vital signs/ or blood pressure/ or body temperature/ or heart rate/ or respiratory rate/ (410477)

122 heart rate*.tw. (159199)

123 pulse rate*.tw. (7614)

124 pulse*.tw. (256042)

125 respiratory rate*.tw. (14992)

126 breathing rate*.tw. (1234)

127 respiration.tw. (67094)

128 breathing.tw. (71424)

129 blood pressure.tw. (294342)

130 systolic.tw. (168584)

131 diastolic.tw. (124777)

132 perfusion.tw. (159943)

133 spo2.tw. (5098)

134 oxygenation.tw. (52011)

135 oxygen saturation.tw. (26170)

136 temperature*.tw. (651316)

137 exp Fever/ (43448)

138 fever*.tw. (171789)

139 hypertherm*.tw. (35327)

140 pyrex*.tw. (5346)

141 Hypothermia/ (13917)

142 hypotherm*.tw. (41950)

143 apyrex*.tw. (210)

144 a-pyrex*.tw. (173)

145 vital sign*.tw. (14891)

146 vitals.tw. (500)

147 vital param*.tw. (1004)

148 vital function*.tw. (2790)

149 early warning score/ (91)

150 (early warning adj3 scor*).tw. (1049)

151 (early warning adj3 system*).tw. (2134)

152 mews.tw. (261)

153 ews.tw. (1986)

154 ewss.tw. (73)

155 (track and trigger).tw. (331)

156 tts.tw. (2485)

157 risk assessment tool*.tw. (3049)

158 febrile.tw. (35432)

159 afebrile.tw. (2897)

160 a-febrile.tw. (2540)

161 121 or 122 or 123 or 124 or 125 or 126 or 127 or 128 or 129 or 130 or 131 or 132 or 133 or 134 or 135 or 136 or 137 or 138 or 139 or 140 or 141 or 142 or 143 or 144 or 145 or 146 or 147 or 148 or 149 or 150 or 151 or 152 or 153 or 154 or 155 or 156 or 157 or 158 or 159 or 160 (2068958)

162 Hospitalization/ (108262)

163 hospital*.tw. (1288499)

164 inhospital.tw. (1750)

165 in-hospital.tw. (89036)

166 admitted.tw. (206110)

167 inpatient*.tw. (108524)

168 surgical.tw. (968814)

169 pacu.tw. (2502)

170 post anaesthesia care unit*.tw. (328)

171 post anesthesia care unit*.tw. (740)

172 postanaesthesia care unit*.tw. (192)

173 postanesthesia care unit*.tw. (1900)

174 icu.tw. (56820)

175 exp Critical Care/ (58129)

176 exp Intensive Care Units/ (85074)

177 intensive care.tw. (144066)

178 itu.tw. (869)

179 aicu.tw. (45)

180 icus.tw. (10393)

181 itus.tw. (43)

182 aicus.tw. (7)

183 pacus.tw. (82)

184 critical care.tw. (27729)

185 (ward or wards).tw. (59542)

186 Triage/ (11887)

187 triage*.tw. (18767)

188 Emergencies/ (40466)

189 (emergency or emergencies).tw. (270872)

190 Recovery Room/ (1297)

191 recovery room*.tw. (3323)

192 adolescent, hospitalized/ or child, hospitalized/ or inpatients/ (29074)

193 162 or 163 or 164 or 165 or 166 or 167 or 168 or 169 or 170 or 171 or 172 or 173 or 174 or 175 or 176 or 177 or 178 or 179 or 180 or 181 or 182 or 183 or 184 or 185 or 186 or 187 or 188 or 189 or 190 or 191 or 192 (2563179)

194 120 and 161 and 193 (5355)

***************************

Database: Embase 1974 to present

Search Strategy: (27/08/20)

--------------------------------------------------------------------------------

1 *ambulatory monitoring/ (3997)

2 ambulatory monitoring/ (11492)

3 ambulatory.tw. (110852)

4 exp *wearable computer/ (1210)

5 exp wearable computer/ (3871)

6 wearable.tw. (13653)

7 (patch or patches).tw. (135225)

8 (body adj3 borne).tw. (86)

9 exp *adhesive agent/ (41931)

10 exp adhesive agent/ (86621)

11 adhesive*.tw. (63227)

12 (sticker* or stick or sticks).tw. (16013)

13 *smart watch/ (54)

14 smart watch/ (130)

15 (watch or watches).tw. (12543)

16 smartwatch*.tw. (370)

17 *telemonitoring/ (1504)

18 telemonitoring/ (3229)

19 telemonitor*.tw. (2331)

20 tele monitor*.tw. (296)

21 exp *telemetry/ (8901)

22 exp telemetry/ (28651)

23 telemet*.tw. (14349)

24 *clothing/ or *shoe/ (7551)

25 clothing/ or shoe/ (20011)

26 cloth*.tw. (19913)

27 *textile/ (2529)

28 textile/ (5748)

29 textile*.tw. (13280)

30 exp *telemedicine/ (21632)

31 exp telemedicine/ (41539)

32 telemedicine*.tw. (14736)

33 biosens*.tw. (41022)

34 *glove/ (1193)

35 glove/ (5534)

36 glove*.tw. (13987)

37 shoe*.tw. (11921)

38 accessor*.tw. (51412)

39 *wrist/ (6462)

40 wrist/ (28141)

41 wrist*.tw. (51874)

42 necklace*.tw. (856)

43 (belt or belts).tw. (14847)

44 armband*.tw. (1161)

45 (shirt or shirts).tw. (1490)

46 contactless.tw. (1765)

47 cableless.tw. (6)

48 exp *sensor/ (46660)

49 exp sensor/ (110298)

50 contact sensor*.tw. (212)

51 unobtrusive.tw. (1919)

52 exp *remote sensing/ (4014)

53 exp remote sensing/ (10697)

54 remote.tw. (85052)

55 *wireless communication/ (2272)

56 wireless communication/ (5355)

57 wireless*.tw. (17980)

58 non contact.tw. (6655)

59 noncontact.tw. (4671)

60 without wires.tw. (25)

61 non-restrict*.tw. (840)

62 unrestrict*.tw. (11317)

63 exp *automation/ (36544)

64 exp automation/ (107074)

65 automat*.tw. (311181)

66 webcam*.tw. (599)

67 web cam*.tw. (259)

68 exp *videorecording/ (16037)

69 exp videorecording/ (88884)

70 video*.tw. (182011)

71 *head-mounted display/ or *wearable computer/ (224)

72 head-mounted display/ or wearable computer/ (501)

73 (body adj3 borne).tw. (86)

74 (head mount* adj3 (display* or device* or system*)).tw. (1297)

75 (head-up adj3 (display* or device* or system*)).tw. (153)

76 (head-worn adj3 (display* or device* or system*)).tw. (31)

77 3 or 4 or 6 or 7 or 8 or 9 or 11 or 12 or 13 or 15 or 16 or 17 or 19 or 20 or 21 or 23 or 24 or 26 or 27 or 29 or 30 or 32 or 33 or 34 or 36 or 37 or 38 or 39 or 41 or 42 or 43 or 44 or 45 or 46 or 47 or 48 or 50 or 51 or 52 or 54 or 55 or 57 or 58 or 59 or 60 or 61 or 62 or 63 or 65 or 66 or 67 or 68 or 69 or 70 or 71 or 73 or 74 or 75 or 76 (1265441)

78 3 or 5 or 6 or 7 or 8 or 10 or 11 or 12 or 14 or 15 or 16 or 18 or 19 or 20 or 22 or 23 or 25 or 26 or 28 or 29 or 31 or 32 or 33 or 35 or 36 or 37 or 38 or 40 or 41 or 42 or 43 or 44 or 45 or 46 or 47 or 49 or 50 or 51 or 53 or 54 or 56 or 57 or 58 or 59 or 60 or 61 or 62 or 64 or 65 or 66 or 67 or 69 or 70 or 72 or 73 or 74 or 75 or 76 (1401398)

79 exp *physiologic monitoring/ (1677)

80 exp physiologic monitoring/ (6455)

81 monitor*.tw. (1108203)

82 exp *oximetry/ (6818)

83 exp oximetry/ (27375)

84 exp *oximeter/ (744)

85 exp oximeter/ (5623)

86 oximet*.tw. (19326)

87 tracker*.tw. (6340)

88 radar*.tw. (4963)

89 *ballistocardiography/ (72)

90 ballistocardiography/ (164)

91 *ballistocardiograph/ (4)

92 ballistocardiograph/ (10)

93 ballisto*.tw. (758)

94 *accelerometry/ (1248)

95 accelerometry/ (7282)

96 *accelerometer/ (2176)

97 accelerometer/ (12337)

98 accelerom*.tw. (21293)

99 gyro*.tw. (4142)

100 *photoelectric plethysmography/ (1491)

101 photoelectric plethysmography/ (4116)

102 (photo* adj3 pleth*).tw. (394)

103 ppg.tw. (5034)

104 photople*.tw. (4390)

105 video pleth*.tw. (6)

106 exp *thermography/ (5787)

107 exp thermography/ (12088)

108 exp *thermograph/ (28)

109 exp thermograph/ (220)

110 thermograph*.tw. (7338)

111 *infrared radiation/ (9112)

112 infrared radiation/ (32272)

113 infrared*.tw. (138691)

114 thermal.tw. (201225)

115 *thermistor/ (227)

116 thermistor/ (1244)

117 thermistor*.tw. (2137)

118 thermoresister*.tw. (0)

119 exp *camera/ (5915)

120 exp camera/ (33767)

121 camera*.tw. (58176)

122 *wavelet analysis/ (1225)

123 wavelet analysis/ (3345)

124 exp *wavelet transform/ (517)

125 exp wavelet transform/ (1315)

126 wavelet*.tw. (13437)

127 *waveform/ (2649)

128 waveform/ (22138)

129 waveform*.tw. (35343)

130 ambient light*.tw. (2049)

131 *piezoelectric lithotripter/ (1)

132 piezoelectric lithotripter/ (5)

133 *piezoelectricity/ (1164)

134 piezoelectricity/ (4002)

135 piezoelectric*.tw. (8397)

136 piezo electric*.tw. (370)

137 exp *impedance/ (8322)

138 exp impedance/ (36592)

139 impedan*.tw. (58031)

140 exp *electrocardiography/ (46538)

141 exp electrocardiography/ (154699)

142 *ambulatory electrocardiography/ (70)

143 ambulatory electrocardiography/ (333)

144 electrocar*.tw. (106621)

145 (ecg or ecgs).tw. (112596)

146 (ekg or ekgs).tw. (8993)

147 electrom*.tw. (104984)

148 electrog*.tw. (26112)

149 exp *electrode/ (28082)

150 exp electrode/ (143152)

151 electrode*.tw. (178982)

152 SensiumVitals.tw. (8)

153 IntelliVue Guardian.tw. (1)

154 VitalPatch.tw. (3)

155 Vitalsolutions.tw. (0)

156 Guardian Angel.tw. (89)

157 Visi Mobile.tw. (6)

158 Current health.tw. (5507)

159 Snap40.tw. (0)

160 Multi-vital ECG patch.tw. (0)

161 Caretaker.tw. (1908)

162 Life scope G3.tw. (0)

163 Biostamp.tw. (11)

164 TAGECG.tw. (0)

165 ZioXT patch.tw. (1)

166 ZioAT patch.tw. (0)

167 Cardea solo.tw. (1)

168 Peerbridge Cor.tw. (0)

169 Radius-7.tw. (49)

170 Radius-PPG.tw. (0)

171 WristOx2.tw. (8)

172 Lifesync.tw. (1)

173 Ariatele.tw. (0)

174 79 or 81 or 82 or 84 or 86 or 87 or 88 or 89 or 91 or 93 or 94 or 96 or 98 or 99 or 100 or 102 or 103 or 104 or 105 or 106 or 108 or 110 or 111 or 113 or 114 or 115 or 117 or 118 or 119 or 121 or 122 or 124 or 126 or 127 or 129 or 130 or 131 or 133 or 135 or 136 or 137 or 139 or 140 or 144 or 145 or 146 or 147 or 148 or 149 or 151 or 152 or 153 or 154 or 155 or 157 or 158 or 159 or 160 or 161 or 162 or 163 or 164 or 165 or 166 or 167 or 168 or 169 or 170 or 171 or 173 or 173 (2028499)

175 80 or 81 or 83 or 85 or 86 or 87 or 88 or 90 or 92 or 93 or 95 or 97 or 98 or 99 or 101 or 102 or 103 or 104 or 105 or 107 or 109 or 110 or 112 or 113 or 114 or 116 or 117 or 118 or 120 or 121 or 123 or 125 or 126 or 128 or 129 or 130 or 132 or 134 or 135 or 136 or 138 or 139 or 141 or 144 or 145 or 146 or 147 or 148 or 150 or 151 or 152 or 153 or 154 or 155 or 157 or 158 or 159 or 160 or 161 or 162 or 163 or 164 or 165 or 166 or 167 or 168 or 169 or 170 or 171 or 173 or 173 (2152615)

176 77 and 174 (225745)

177 1 or 142 or 176 (227345)

178 78 and 175 (258920)

179 2 or 143 or 178 (263881)

180 *vital sign/ (1327)

181 vital sign/ (23653)

182 *blood pressure/ or *diastolic blood pressure/ or *systolic blood pressure/ (87175)

183 blood pressure/ or diastolic blood pressure/ or systolic blood pressure/ (388166)

184 exp *body temperature/ (11566)

185 exp body temperature/ (52272)

186 exp *heart rate/ (62075)

187 exp heart rate/ (249777)

188 *breathing rate/ (3707)

189 breathing rate/ (39825)

190 heart rate*.tw. (216870)

191 pulse rate*.tw. (10676)

192 pulse*.tw. (296797)

193 respiratory rate*.tw. (22895)

194 breathing rate*.tw. (1690)

195 respiration.tw. (73143)

196 breathing.tw. (102148)

197 blood pressure.tw. (417971)

198 systolic.tw. (270994)

199 diastolic.tw. (193498)

200 perfusion.tw. (215909)

201 spo2.tw. (11601)

202 *pulse rate/ (3451)

203 pulse rate/ (37412)

204 oxygenation.tw. (69563)

205 oxygen saturation.tw. (39081)

206 temperature*.tw. (666834)

207 exp *fever/ (22703)

208 exp fever/ (238988)

209 fever*.tw. (228572)

210 *hypothermia/ (14402)

211 hypothermia/ (33156)

212 *hyperthermia/ (10636)

213 hyperthermia/ (21744)

214 hypotherm*.tw. (48982)

215 hypertherm*.tw. (42665)

216 apyrex*.tw. (411)

217 a-pyrex*.tw. (243)

218 pyrex*.tw. (8701)

219 vital sign*.tw. (29345)

220 vitals.tw. (3388)

221 vital param*.tw. (1712)

222 vital function*.tw. (3595)

223 *early warning score/ (128)

224 early warning score/ (244)

225 (early warning adj3 scor*).tw. (1816)

226 (early warning adj3 system*).tw. (2743)

227 mews.tw. (491)

228 ews.tw. (2990)

229 ewss.tw. (89)

230 (track and trigger).tw. (525)

231 tts.tw. (4262)

232 risk assessment tool*.tw. (4743)

233 febrile.tw. (55234)

234 afebrile.tw. (6571)

235 a-febrile.tw. (3575)

236 180 or 182 or 184 or 186 or 188 or 190 or 191 or 192 or 193 or 194 or 195 or 196 or 197 or 198 or 199 or 200 or 201 or 202 or 204 or 205 or 206 or 207 or 209 or 210 or 212 or 214 or 215 or 216 or 217 or 218 or 219 or 220 or 221 or 222 or 223 or 225 or 226 or 227 or 228 or 229 or 230 or 231 or 232 or 233 or 234 or 235 (2374398)

237 181 or 183 or 185 or 187 or 189 or 190 or 191 or 192 or 193 or 194 or 195 or 196 or 197 or 198 or 199 or 200 or 201 or 203 or 204 or 205 or 206 or 208 or 209 or 211 or 213 or 214 or 215 or 216 or 217 or 218 or 219 or 220 or 221 or 222 or 224 or 225 or 226 or 227 or 228 or 229 or 230 or 231 or 232 or 233 or 234 or 235 (2619139)

238 *hospitalization/ (34982)

239 hospitalization/ (376370)

240 hospital*.tw. (1960850)

241 inhospital.tw. (5811)

242 in-hospital.tw. (143939)

243 admitted.tw. (360252)

244 inpatient*.tw. (181329)

245 exp *hospital patient/ (29516)

246 exp hospital patient/ (178432)

247 surgical.tw. (1286932)

248 pacu.tw. (4203)

249 *recovery room/ (984)

250 recovery room/ (6518)

251 recovery room*.tw. (4549)

252 post anaesthesia care unit*.tw. (492)

253 post anesthesia care unit*.tw. (1238)

254 postanaesthesia care unit*.tw. (233)

255 postanesthesia care unit*.tw. (2060)

256 *intensive care/ (62700)

257 intensive care/ (124945)

258 *intensive care unit/ (34302)

259 intensive care unit/ (155944)

260 icu.tw. (117477)

261 intensive care.tw. (210656)

262 itu.tw. (2456)

263 aicu.tw. (107)

264 icus.tw. (17007)

265 itus.tw. (105)

266 aicus.tw. (10)

267 pacus.tw. (96)

268 critical care.tw. (46988)

269 (ward or wards).tw. (89962)

270 exp *ward/ (85082)

271 exp ward/ (367484)

272 exp *emergency health service/ (47431)

273 exp emergency health service/ (102497)

274 triage*.tw. (29343)

275 exp *emergency/ (13589)

276 exp emergency/ (53011)

277 (emergency or emergencies).tw. (395565)

278 exp *hospital patient/ (29516)

279 exp hospital patient/ (178432)

280 238 or 240 or 241 or 242 or 243 or 244 or 245 or 247 or 248 or 249 or 251 or 252 or 253 or 254 or 255 or 256 or 258 or 260 or 261 or 262 or 263 or 264 or 265 or 266 or 267 or 268 or 269 or 270 or 272 or 274 or 275 or 277 or 278 (3603118)

281 239 or 240 or 241 or 242 or 243 or 244 or 246 or 247 or 248 or 250 or 251 or 252 or 253 or 254 or 255 or 257 or 259 or 260 or 261 or 262 or 263 or 264 or 265 or 266 or 267 or 268 or 269 or 271 or 273 or 274 or 276 or 277 or 279 (3786867)

282 177 and 236 and 280 (8780)

283 179 and 237 and 281 (10492)

284 conference*.pt. (4619461)

285 282 not 284 (4494)

***************************

CINAHL (27/08/20)

| **#** | **Query** |
| --- | --- |
| 1 | (MH "Blood Pressure Monitoring, Ambulatory") |
| 2 | (MH "Electrocardiography, Ambulatory") |
| 3 | TI ambulatory OR AB ambulatory |
| 4 | TI wearable OR AB wearable |
| 5 | TI ( patch or patches ) OR AB ( patch or patches ) |
| 6 | TI body n3 borne OR AB body n3 borne |
| 7 | TI ( "head mount*" n3 (display* or device* or system*) ) OR AB ( "head mount*" n3 (display* or device* or system*) ) |
| 8 | TI ( "head up" n3 (display* or device* or system*) ) OR AB ( "head up" n3 (display* or device* or system*) ) |
| 9 | TI ( "head worn" n3 (display* or device* or system*) ) OR AB ( "head worn" n3 (display* or device* or system*) ) |
| 10 | TI telemedicine* OR AB telemedicine* |
| 11 | (MH "Biosensing Techniques+") |
| 12 | TI telemet* OR AB telemet* |
| 13 | TI biosens* OR AB biosens* |
| 14 | TI glove* OR AB glove* |
| 15 | TI shoe* OR AB shoe* |
| 16 | TI adhesive* OR AB adhesive* |
| 17 | TI accessor* OR AB accessor* |
| 18 | (MH "Wrist") |
| 19 | TI wrist* OR AB wrist* |
| 20 | TI necklace* OR AB necklace* |
| 21 | TI ( belt or belts ) OR AB ( belt or belts ) |
| 22 | TI armband* OR AB armband* |
| 23 | (MH "Clothing") OR (MH "Shoes+") |
| 24 | TI ( shirt or shirts ) OR AB ( shirt or shirts ) |
| 25 | TI contactless OR AB contactless |
| 26 | TI cableless OR AB cableless |
| 27 | (MH "Wearable Sensors") |
| 28 | TI ( sticker* or stick or sticks ) OR AB ( sticker* or stick or sticks ) |
| 29 | TI "contact sensor*" OR AB "contact sensor*" |
| 30 | TI unobtrusive OR AB unobtrusive |
| 31 | TI remote OR AB remote |
| 32 | (MH "Wireless Communications") |
| 33 | TI wireless* OR AB wireless* |
| 34 | (MH "Adhesives") |
| 35 | TI "non contact" OR AB "non contact" |
| 36 | TI noncontact OR AB noncontact |
| 37 | TI "without wires" OR AB "without wires" |
| 38 | TI non-restrict* OR AB non-restrict* |
| 39 | TI unrestrict* OR AB unrestrict* |
| 40 | TI ( watch or watches ) OR AB ( watch or watches ) |
| 41 | (MH "Automation+") |
| 42 | TI automat* OR AB automat* |
| 43 | TI webcam* OR AB webcam* |
| 44 | TI "web cam*" OR AB "web cam*" |
| 45 | TI cloth* OR AB cloth* |
| 46 | (MH "Videorecording") |
| 47 | TI video* OR AB video* |
| 48 | (MH "Textiles") |
| 49 | TI textile* OR AB textile* |
| 50 | (MH "Telemedicine") |
| 51 | (MH "Telenursing") |
| 52 | TI telemonitor* OR AB telemonitor* |
| 53 | TI "tele monitor*" OR AB "tele monitor*" |
| 54 | TI smartwatch* OR AB smartwatch* |
| 55 | S3 OR S4 OR S5 OR S6 OR S7 OR S8 OR S9 OR S10 OR S11 OR S12 OR S13 OR S14 OR S15 OR S16 OR S17 OR S18 OR S19 OR S20 OR S21 OR S22 OR S23 OR S24 OR S25 OR S26 OR S27 OR S28 OR S29 OR S30 OR S31 OR S32 OR S33 OR S34 OR S35 OR S36 OR S37 OR S38 OR S39 OR S40 OR S41 OR S42 OR S43 OR S44 OR S45 OR S46 OR S47 OR S48 OR S49 OR S50 OR S51 OR S52 OR S53 OR S54 |
| 56 | (MH "Monitoring, Physiologic+/MT") |
| 57 | TI monitor* OR AB monitor* |
| 58 | (MH "Oximetry+") |
| 59 | TI oximet* OR AB oximet* |
| 60 | TI tracker* OR AB tracker* |
| 61 | TI radar* OR AB radar* |
| 62 | TI ballisto* OR AB ballisto* |
| 63 | (MH "Accelerometry+") |
| 64 | TI accelerom* OR AB accelerom* |
| 65 | TI gyro* OR AB gyro* |
| 66 | TI photople* OR AB photople* |
| 67 | TI ppg OR AB ppg |
| 68 | TI videopleth* OR AB videopleth* |
| 69 | TI "video pleth*" OR AB "video pleth*" |
| 70 | TI photo* n3 pleth* OR AB photo* n3 pleth* |
| 76 | TI thermoresister* OR AB thermoresister* |
| 77 | TI camera* OR AB camera* |
| 78 | TI wavelet* OR AB wavelet* |
| 79 | (MH "Waveforms") |
| 80 | TI waveform* OR AB waveform* |
| 81 | TI "ambient light*" OR AB "ambient light*" |
| 82 | TI piezoelectric* OR AB piezoelectric* |
| 83 | TI "piezo electric*" OR AB "piezo electric*" |
| 84 | TI impedan* OR AB impedan* |
| 85 | (MH "Electric Impedance") |
| 86 | (MH "Electrocardiography+") |
| 87 | TI electrocar* OR AB electrocar* |
| 88 | TI ( ecg or ecgs ) OR AB ( ecg or ecgs ) |
| 89 | TI ( ekg or ekgs ) OR AB ( ekg or ekgs ) |
| 90 | TI electrom* OR AB electrom* |
| 91 | TI electrog* OR AB electrog* |
| 92 | (MH "Electrodes") |
| 93 | TI electrode* OR AB electrode* |
| 94 | TI SensiumVitals OR AB SensiumVitals |
| 95 | TI IntelliVue Guardian OR AB IntelliVue Guardian |
| 96 | TI VitalPatch OR AB VitalPatch |
| 97 | TI Vitalsolutions OR AB Vitalsolutions |
| 98 | TI "Guardian Angel" OR AB "Guardian Angel" |
| 99 | TI "Visi Mobile" OR AB "Visi Mobile" |
| 100 | TI "Current health" OR AB "Current health" |
| 101 | TI Snap40 OR AB Snap40 |
| 102 | TI "Multi-vital ECG patch" OR AB "Multi-vital ECG patch" |
| 103 | TI Caretaker OR AB Caretaker |
| 104 | TI "Life scope G3" OR AB "Life scope G3" |
| 105 | TI Biostamp OR AB Biostamp |
| 106 | TI TAGECG OR AB TAGECG |
| 107 | TI "ZioXT patch" OR AB "ZioXT patch" |
| 108 | TI "ZioAT patch" OR AB "ZioAT patch" |
| 109 | TI "Cardea solo" OR AB "Cardea solo" |
| 110 | TI "Peerbridge Cor" OR AB "Peerbridge Cor" |
| 111 | TI Radius-7 OR AB Radius-7 |
| 112 | TI Radius-PPG OR AB Radius-PPG |
| 113 | TI WristOx2 OR AB WristOx2 |
| 114 | TI Lifesync OR AB Lifesync |
| 115 | TI Ariatele OR AB Ariatele |
| 116 | S56 OR S57 OR S58 OR S59 OR S60 OR S61 OR S62 OR S63 OR S64 OR S65 OR S66 OR S67 OR S68 OR S69 OR S70 OR S71 OR S72 OR S73 OR S74 OR S75 OR S76 OR S77 OR S78 OR S79 OR S80 OR S81 OR S82 OR S83 OR S84 OR S85 OR S86 OR S87 OR S88 OR S89 OR S90 OR S91 OR S92 OR S93 OR S94 OR S95 OR S96 OR S97 OR S98 OR S99 OR S100 OR S101 OR S102 OR S103 OR S104 OR S105 OR S106 OR S107 OR S108 OR S109 OR S110 OR S111 OR S112 OR S113 OR S114 OR S115 |
| 117 | S55 AND S116 |
| 118 | S1 OR S2 OR S117 |
| 119 | (MH "Blood Pressure Determination") OR (MH "Body Temperature Determination") OR (MH "Pulse+") OR (MH "Respiratory Rate") OR (MH "Vital Signs") |
| 120 | TI "heart rate*" OR AB "heart rate*" |
| 121 | TI "pulse rate*" OR AB "pulse rate*" |
| 122 | TI pulse* OR AB pulse* |
| 123 | TI "respiratory rate*" OR AB "respiratory rate*" |
| 124 | TI "breathing rate*" OR AB "breathing rate*" |
| 125 | TI respiration OR AB respiration |
| 126 | TI breathing OR AB breathing |
| 127 | TI "blood pressure" OR AB "blood pressure" |
| 128 | TI systolic OR AB systolic |
| 129 | TI diastolic OR AB diastolic |
| 130 | TI perfusion OR AB perfusion |
| 131 | TI spo2 OR AB spo2 |
| 132 | TI oxygenation OR AB oxygenation |
| 133 | TI "oxygen saturation" OR AB "oxygen saturation" |
| 134 | TI temperature* OR AB temperature* |
| 135 | (MH "Fever+") |
| 136 | TI fever* OR AB fever* |
| 137 | (MH "Hypothermia") |
| 138 | TI hypertherm* OR AB hypertherm* |
| 139 | TI pyrex* OR AB pyrex* |
| 140 | TI hypotherm* OR AB hypotherm* |
| 141 | TI apyrex* OR AB apyrex* |
| 142 | TI a-pyrex* OR AB a-pyrex* |
| 143 | TI "vital sign*" OR AB "vital sign*" |
| 144 | TI vitals OR AB vitals |
| 145 | TI "vital param*" OR AB "vital param*" |
| 146 | TI "vital function*" OR AB "vital function*" |
| 147 | (MH "Early Warning Score") |
| 148 | TI "early warning" n3 scor* OR AB "early warning" n3 scor* |
| 149 | TI "early warning" n3 system* OR AB "early warning" n3 system* |
| 150 | TI mews OR AB mews |
| 151 | TI ews OR AB ews |
| 152 | TI ewss OR AB ewss |
| 153 | TI ( "track and trigger" ) OR AB ( "track and trigger" ) |
| 154 | TI tts OR AB tts |
| 155 | TI "risk assessment tool*" OR AB "risk assessment tool*" |
| 156 | TI febrile OR AB febrile |
| 157 | TI afebrile OR AB afebrile |
| 158 | TI "a-febrile" OR AB "a-febrile" |
| 159 | S119 OR S120 OR S121 OR S122 OR S123 OR S124 OR S125 OR S126 OR S127 OR S128 OR S129 OR S130 OR S131 OR S132 OR S133 OR S134 OR S135 OR S136 OR S137 OR S138 OR S139 OR S140 OR S141 OR S142 OR S143 OR S144 OR S145 OR S146 OR S147 OR S148 OR S149 OR S150 OR S151 OR S152 OR S153 OR S154 OR S155 OR S156 OR S157 OR S158 |
| 160 | MH "Hospitalization" |
| 161 | TI hospital* OR AB hospital* |
| 162 | TI inhospital OR AB inhospital |
| 163 | TI "in-hospital" OR AB "in-hospital" |
| 164 | (MH "Inpatients") |
| 165 | (MH "Adolescent, Hospitalized") OR (MH "Aged, Hospitalized") OR (MH "Child, Hospitalized") OR (MH "Infant, Hospitalized") |
| 166 | TI admitted OR AB admitted |
| 167 | TI inpatient* OR AB inpatient* |
| 168 | TI surgical OR AB surgical |
| 169 | (MH "Intensive Care Units") OR (MH "Post Anesthesia Care Units") |
| 170 | TI pacu OR AB pacu |
| 171 | TI "post anaesthesia care unit*" OR AB "post anaesthesia care unit*" |
| 172 | TI "post anesthesia care unit*" OR AB "post anesthesia care unit*" |
| 173 | TI "postanaesthesia care unit*" OR AB "postanaesthesia care unit*" |
| 174 | TI "postanesthesia care unit*" OR AB "postanesthesia care unit*" |
| 175 | TI icu OR AB icu |
| 176 | TI aicu OR AB aicu |
| 177 | TI icus OR AB icus |
| 178 | TI itus OR AB itus |
| 179 | TI pacus OR AB pacus |
| 180 | TI "intensive care" OR AB "intensive care" |
| 181 | (MH "Critical Care") |
| 182 | TI "critical care" OR AB "critical care" |
| 183 | TI ( ward or wards ) OR AB ( ward or wards ) |
| 184 | (MH "Triage") |
| 185 | TI triage* OR AB triage* |
| 186 | (MH "Emergencies+") |
| 187 | TI ( emergency or emergencies ) OR AB ( emergency or emergencies ) |
| 188 | (MH "Emergency Medical Services+") |
| 189 | TI "recovery room*" OR AB "recovery room*" |
| 190 | S160 OR S161 OR S162 OR S163 OR S164 OR S165 OR S166 OR S167 OR S168 OR S169 OR S170 OR S171 OR S172 OR S173 OR S174 OR S175 OR S176 OR S177 OR S178 OR S179 OR S180 OR S181 OR S182 OR S183 OR S184 OR S185 OR S186 OR S187 OR S188 OR S189 |
| 191 | S118 AND S159 AND S190 |

COCHRANE LIBRARY

Search Name: ambulatory monitoring

Last Saved: 27/08/2020 14:05:22

Comment:

ID Search

#1 MeSH descriptor: [Monitoring, Ambulatory] explode all trees

#2 ambulatory:ti,ab,kw

#3 MeSH descriptor: [Wearable Electronic Devices] explode all trees

#4 wearable:ti,ab,kw

#5 (body near/3 borne):ti,ab,kw

#6 ("head mount*" near/3 (display* or device* or system*)):ti,ab,kw

#7 (head-up near/3 (display* or device* or system*)):ti,ab,kw

#8 (head-worn near/3 (display* or device* or system*)):ti,ab,kw

#9 (patch or patches):ti,ab,kw

#10 MeSH descriptor: [Adhesives] this term only

#11 adhesive*:ti,ab,kw

#12 (sticker* or stick or sticks):ti,ab,kw

#13 (watch or watches):ti,ab,kw

#14 smartwatch*:ti,ab,kw

#15 telemonitor*:ti,ab,kw

#16 "tele monitor*":ti,ab,kw

#17 MeSH descriptor: [Telemetry] explode all trees

#18 telemet*:ti,ab,kw

#19 MeSH descriptor: [Clothing] this term only

#20 MeSH descriptor: [Shoes] this term only

#21 cloth*:ti,ab,kw

#22 MeSH descriptor: [Textiles] explode all trees

#23 textile*:ti,ab,kw

#24 MeSH descriptor: [Telemedicine] explode all trees

#25 telemedicine*:ti,ab,kw

#26 MeSH descriptor: [Biosensing Techniques] explode all trees

#27 biosens*:ti,ab,kw

#28 glove*:ti,ab,kw

#29 shoe*:ti,ab,kw

#30 accessor*:ti,ab,kw

#31 MeSH descriptor: [Wrist] this term only

#32 wrist*:ti,ab,kw

#33 necklace*:ti,ab,kw

#34 (belt or belts):ti,ab,kw

#35 armband*:ti,ab,kw

#36 (shirt or shirts):ti,ab,kw

#37 contactless:ti,ab,kw

#38 cableless:ti,ab,kw

#39 "contact sensor*":ti,ab,kw

#40 unobtrusive:ti,ab,kw

#41 remote:ti,ab,kw

#42 MeSH descriptor: [Wireless Technology] this term only

#43 wireless*:ti,ab,kw

#44 "non contact":ti,ab,kw

#45 noncontact:ti,ab,kw

#46 "without wires":ti,ab,kw

#47 non-restrict*:ti,ab,kw

#48 unrestrict*:ti,ab,kw

#49 MeSH descriptor: [Automation] this term only

#50 automat*:ti,ab,kw

#51 webcam*:ti,ab,kw

#52 web-cam*:ti,ab,kw

#53 video*:ti,ab,kw

#54 #1 or #2 or #3 or #4 or #5 or #6 or #7 or #8 or #9 or #10 or #11 or #12 or #13 or #14 or #15 or #16 or #17 or #18 or #19 or #20 or #21 or #22 or #23 or #24 or #25 or #26 or #27 or #28 or #29 or #30 or #31 or #32 or #33 or #34 or #35 or #36 or #37 or #38 or #39 or #40 or #41 or #42 or #43 or #44 or #45 or #46 or #47 or #48 or #49 or #50 or #51 or #52 or #53

#55 MeSH descriptor: [Monitoring, Physiologic] this term only

#56 monitor*:ti,ab,kw

#57 MeSH descriptor: [Oximetry] this term only

#58 oximet*:ti,ab,kw

#59 tracker*:ti,ab,kw

#60 MeSH descriptor: [Radar] this term only

#61 radar*:ti,ab,kw

#62 MeSH descriptor: [Ballistocardiography] this term only

#63 ballisto*:ti,ab,kw

#64 MeSH descriptor: [Accelerometry] explode all trees

#65 accelerom*:ti,ab,kw

#66 gyro*:ti,ab,kw

#67 MeSH descriptor: [Photoplethysmography] this term only

#68 ppg:ti,ab,kw

#69 photople*:ti,ab,kw

#70 videopleth*:ti,ab,kw

#71 "video pleth*":ti,ab,kw

#72 (photo* near/3 pleth*):ti,ab,kw

#73 MeSH descriptor: [Thermography] this term only

#74 thermograph*:ti,ab,kw

#75 MeSH descriptor: [Infrared Rays] this term only

#76 infrared*:ti,ab,kw

#77 thermal:ti,ab,kw

#78 thermistor*:ti,ab,kw

#79 thermoresister*:ti,ab,kw

#80 camera*:ti,ab,kw

#81 MeSH descriptor: [Wavelet Analysis] this term only

#82 wavelet*:ti,ab,kw

#83 waveform*:ti,ab,kw

#84 "ambient light*":ti,ab,kw

#85 piezoelectric*:ti,ab,kw

#86 "piezo electric*":ti,ab,kw

#87 MeSH descriptor: [Electric Impedance] this term only

#88 impedan*:ti,ab,kw

#89 MeSH descriptor: [Electrocardiography] explode all trees

#90 electrocar*:ti,ab,kw

#91 (ecg or ecgs):ti,ab,kw

#92 (ekg or ekgs):ti,ab,kw

#93 electrom*:ti,ab,kw

#94 electrog*:ti,ab,kw

#95 MeSH descriptor: [Electrodes] explode all trees

#96 electrode*:ti,ab,kw

#97 SensiumVitals:ti,ab,kw

#98 "IntelliVue Guardian":ti,ab,kw

#99 VitalPatch:ti,ab,kw

#100 Vitalsolutions:ti,ab,kw

#101 "Guardian Angel":ti,ab,kw

#102 "Visi Mobile":ti,ab,kw

#103 "Current health":ti,ab,kw

#104 Snap40:ti,ab,kw

#105 "Multi-vital ECG patch":ti,ab,kw

#106 Caretaker:ti,ab,kw

#107 "Life scope G3":ti,ab,kw

#108 Biostamp:ti,ab,kw

#109 TAGECG:ti,ab,kw

#110 "ZioXT patch":ti,ab,kw

#111 "ZioAT patch":ti,ab,kw

#112 "Cardea solo":ti,ab,kw

#113 "Peerbridge Cor":ti,ab,kw

#114 "Radius-7":ti,ab,kw

#115 "Radius-PPG":ti,ab,kw

#116 WristOx2:ti,ab,kw

#117 Lifesync:ti,ab,kw

#118 Ariatele:ti,ab,kw

#119 #55 or #56 or #57 or #58 or #59 or #60 or #61 or #62 or #63 or #64 or #65 or #66 or #67 or #68 or #69 or #70 or #71 or #72 or #73 or #74 or #75 or #76 or #77 or #78 or #79 or #80 or #81 or #82 or #83 or #84 or #85 or #86 or #87 or #88 or #89 or #90 or #91 or #92 or #93 or #94 or #95 or #96 or #97 or #98 or #99 or #100 or #101 or #102 or #103 or #104 or #105 or #106 or #107 or #108 or #109 or #110 or #111 or #112 or #113 or #114 or #115 or #116 or #117 or #118

#120 #54 and #119

#121 #1 or #120

#122 MeSH descriptor: [Vital Signs] explode all trees

#123 "heart rate*":ti,ab,kw

#124 "pulse rate*":ti,ab,kw

#125 pulse*:ti,ab,kw

#126 "respiratory rate*":ti,ab,kw

#127 "breathing rate*":ti,ab,kw

#128 respiration:ti,ab,kw

#129 breathing:ti,ab,kw

#130 "blood pressure":ti,ab,kw

#131 systolic:ti,ab,kw

#132 diastolic:ti,ab,kw

#133 perfusion:ti,ab,kw

#134 spo2:ti,ab,kw

#135 oxygenation:ti,ab,kw

#136 oxygen saturation:ti,ab,kw

#137 temperature*:ti,ab,kw

#138 MeSH descriptor: [Fever] explode all trees

#139 fever*:ti,ab,kw

#140 hypertherm*:ti,ab,kw

#141 pyrex*:ti,ab,kw

#142 MeSH descriptor: [Hypothermia] this term only

#143 hypotherm*:ti,ab,kw

#144 apyrex*:ti,ab,kw

#145 a-pyrex*:ti,ab,kw

#146 "vital sign*":ti,ab,kw

#147 vitals:ti,ab,kw

#148 "vital param*":ti,ab,kw

#149 "vital function*":ti,ab,kw

#150 MeSH descriptor: [Early Warning Score] this term only

#151 ("early warning" near/3 scor*):ti,ab,kw

#152 ("early warning" near/3 system*):ti,ab,kw

#153 mews:ti,ab,kw

#154 ews:ti,ab,kw

#155 ewss:ti,ab,kw

#156 "track and trigger":ti,ab,kw

#157 tts:ti,ab,kw

#158 "risk assessment tool*":ti,ab,kw

#159 febrile:ti,ab,kw

#160 afebrile:ti,ab,kw

#161 a-febrile:ti,ab,kw

#162 #122 or #123 or #124 or #125 or #126 or #127 or #128 or #129 or #130 or #131 or #132 or #133 or #134 or #135 or #136 or #137 or #138 or #139 or #140 or #141 or #142 or #143 or #144 or #145 or #146 or #147 or #148 or #149 or #150 or #151 or #152 or #153 or #154 or #155 or #156 or #157 or #158 or #159 or #160 or #161

#163 MeSH descriptor: [Hospitalization] this term only

#164 hospital*:ti,ab,kw

#165 inhospital:ti,ab,kw

#166 in-hospital:ti,ab,kw

#167 admitted:ti,ab,kw

#168 inpatient*:ti,ab,kw

#169 surgical:ti,ab,kw

#170 pacu:ti,ab,kw

#171 "post anaesthesia care unit*":ti,ab,kw

#172 "post anesthesia care unit*":ti,ab,kw

#173 "postanaesthesia care unit*":ti,ab,kw

#174 "postanesthesia care unit*":ti,ab,kw

#175 icu:ti,ab,kw

#176 MeSH descriptor: [Critical Care] explode all trees

#177 MeSH descriptor: [Intensive Care Units] explode all trees

#178 "intensive care":ti,ab,kw

#179 itu:ti,ab,kw

#180 aicu:ti,ab,kw

#181 icus:ti,ab,kw

#182 itus:ti,ab,kw

#183 aicus:ti,ab,kw

#184 pacus:ti,ab,kw

#185 "critical care":ti,ab,kw

#186 (ward or wards):ti,ab,kw

#187 MeSH descriptor: [Triage] this term only

#188 triage*:ti,ab,kw

#189 MeSH descriptor: [Emergencies] this term only

#190 (emergency or emergencies):ti,ab,kw

#191 MeSH descriptor: [Recovery Room] this term only

#192 "recovery room*":ti,ab,kw

#193 MeSH descriptor: [Adolescent, Hospitalized] this term only

#194 MeSH descriptor: [Child, Hospitalized] this term only

#195 MeSH descriptor: [Inpatients] this term only

#196 #163 or #164 or #165 or #166 or #167 or #168 or #169 or #170 or #171 or #172 or #173 or #174 or #175 or #176 or #177 or #178 or #179 or #180 or #181 or #182 or #183 or #184 or #185 or #186 or #187 or #188 or #189 or #190 or #191 or #192 or #193 or #194 or #195

#197 #121 and #162 and #196

2,526 (23 Cochrane Systematic reviews & 2,503 trials-CENTRAL)

HTA (10/09/20)

“any field”

Ambulatory AND monitor*

OR

Vital sign* AND monitor*
