# Supplementary material for: The impact of wearable continuous vital sign monitoring on deterioration detection and clinical outcomes in hospitalised patients: a systematic review and meta-analysis
Source: Crit Care. 2021 Sep 28;25:351. doi: 10.1186/s13054-021-03766-4 (PMC8477465; doi:10.1186/s13054-021-03766-4)
Supplement: Supplementary file 3 — Additional file 3. Final Search strategies—registries [file 13054_2021_3766_MOESM3_ESM.docx]

# Appendix 3 – Final Search strategies – registries

ClinicalTrials.gov (10/09/20)

Advanced search/ Targeted search- Intervention/treatment:

912 Studies found for: **(ambulatory AND monitoring) OR (vital signs AND monitoring)**

ISRCTN Registry (10/09/20)

Advanced search/Text search

Search 1= “vital signs” monitoring 157 results

Search 2= “ambulatory monitoring” 15 results
