# Supplementary material for: The impact of wearable continuous vital sign monitoring on deterioration detection and clinical outcomes in hospitalised patients: a systematic review and meta-analysis
Source: Crit Care. 2021 Sep 28;25:351. doi: 10.1186/s13054-021-03766-4 (PMC8477465; doi:10.1186/s13054-021-03766-4)
Supplement: Supplementary file 4 — Additional file 4. Final Search strategies—grey literature [file 13054_2021_3766_MOESM4_ESM.docx]

# Appendix 4 – Final Search strategies – grey literature

**MedRxiv –** search 21^st^ December 2020.

Search terms used ordered by Newest first and selected the first 50, then by Best Match and selected within the first 50:

Ambulatory monitoring (100 selected out of 274)

“Vital signs” monitoring (24 selected out of 24)

Wearable monitoring (100 selected out of 149)

170/224 results after removing duplicates

**Google scholar** – search 8^th^ January 2021

Search filtered not to include patents and sorted by date and selected the first 50 records then by relevance and selected the first 50:

Ambulatory monitoring (50 selected out of 921000 results, sorted by relevance. 50 selected out of 1220 results, sorted by date)

“Vital signs” monitoring (50 selected out of 454000 results, sorted by relevance. 50 selected out of 1220 results, sorted by date)

Wearable monitoring (50 selected out of 342000 results, sorted by relevance. 50 selected out of 867 results, sorted by date)

240/300 results after removing duplicates
