# Supplementary material for: The impact of wearable continuous vital sign monitoring on deterioration detection and clinical outcomes in hospitalised patients: a systematic review and meta-analysis
Source: Crit Care. 2021 Sep 28;25:351. doi: 10.1186/s13054-021-03766-4 (PMC8477465; doi:10.1186/s13054-021-03766-4)
Supplement: Supplementary file 5 — Additional file 5. GradePro tables [file 13054_2021_3766_MOESM5_ESM.docx]

# Appendix 5 – GradePro tables

| **Certainty assessment** | | | | | | | **№ of patients** | | **Effect** | | **Certainty** | **Importance** |
| --- | --- | --- | --- | --- | --- | --- | --- | --- | --- | --- | --- | --- |
| **№ of studies** | **Study design** | **Risk of bias** | **Inconsistency** | **Indirectness** | **Imprecision** | **Other considerations** | **WMS (with or without manual measurements)** | **intermittent monitoring** | **Relative (95% CI)** | **Absolute (95% CI)** |  |  |
| **Intensive care transfers - RCTs and cluster RCT** | | | | | | | | | | | | |
| 2 | randomised trials | serious ^b^ | serious ^f^ | not serious | serious ^d^ | strong association | 4/200 (2.0%) | 7/151 (4.6%) | **RR 0.50** (0.12 to 2.07) | **23 fewer per 1,000** (from 41 fewer to 50 more) | ⨁⨁◯◯ LOW |  |
| **Intensive care transfers - Before-after studies** | | | | | | | | | | | | |
| 3 | observational studies | serious ^a^ | not serious | not serious | serious ^d^ | publication bias strongly suspected ^e^ | 88/1698 (5.2%) | 90/1516 (5.9%) | **RR 0.90** (0.66 to 1.22) | **6 fewer per 1,000** (from 20 fewer to 13 more) | ⨁◯◯◯ VERY LOW |  |
| **Rapid response or cardiac arrest team call – Before-after studies** | | | | | | | | | | | | |
| 3 | observational studies | serious ^a^ | not serious | not serious | serious ^d^ | publication bias strongly suspected ^e^ | 176/1698 (10.4%) | 176/1516 (11.6%) | **RR 0.84** (0.69 to 1.01) | **19 fewer per 1,000** (from 36 fewer to 1 more) | ⨁◯◯◯ VERY LOW |  |
| **All complications prevalence - RCTs and cluster RCT** | | | | | | | | | | | | |
| 3 | randomised trials | serious ^g^ | serious ^f^ | not serious | serious ^d^ | strong association | 155/296 (52.4%) | 109/250 (43.6%) | **RR 0.96** (0.29 to 3.15) | **17 fewer per 1,000** (from 310 fewer to 937 more) | ⨁⨁◯◯ LOW |  |
| **All complications prevalence - Before-after studies** | | | | | | | | | | | | |
| 2 | observational studies | serious ^a^ | not serious | not serious | serious ^d^ | publication bias strongly suspected ^e^ | 47/541 (8.7%) | 159/665 (23.9%) | **RR 0.43** (0.18 to 1.03) | **136 fewer per 1,000** (from 196 fewer to 7 more) | ⨁◯◯◯ VERY LOW |  |
| **Major complications prevalence - RCTs and cluster RCT** | | | | | | | | | | | | |
| 3 | randomised trials | serious ^g^ | serious ^f^ | not serious | serious ^d^ | none | 11/296 (3.7%) | 17/250 (6.8%) | **RR 0.53** (0.23 to 1.22) | **32 fewer per 1,000** (from 52 fewer to 15 more) | ⨁◯◯◯ VERY LOW |  |

**CI:** Confidence interval; **RR:** Risk ratio

#### Explanations

a. Moderate risk of bias in ROBINS-I

b. Some concerns on ROB2 in one of the studies

c. CI cross the clinical decision threshold

d. Suspected reporting bias (see ROBINS-I score)

e. One of the studies is a cluster RCT

f. Some concerns on ROB2 in the 2 studies

| **Certainty assessment** | | | | | | | **№ of patients** | | **Effect** | | **Certainty** | **Importance** |
| --- | --- | --- | --- | --- | --- | --- | --- | --- | --- | --- | --- | --- |
| **№ of studies** | **Study design** | **Risk of bias** | **Inconsistency** | **Indirectness** | **Imprecision** | **Other considerations** | **WMS (with our without standard care)** | **standard care** | **Relative (95% CI)** | **Absolute (95% CI)** |  |  |
| **Mortality - RCTs and cluster RCT** | | | | | | | | | | | | |
| 3 | randomised trials | serious ^a^ | serious ^b^ | not serious | serious ^c^ | none | 2/296 (0.7%) | 0/250 (0.0%) | **OR 2.48** (0.25 to 24.07) | **0 fewer per 1,000** (from 0 fewer to 0 fewer) | ⨁◯◯◯ VERY LOW |  |
| **Mortality - Before-after studies** | | | | | | | | | | | | |
| 3 | observational studies | serious ^d^ | not serious | not serious | serious ^c^ | publication bias strongly suspected ^e^ | 4/1698 (0.2%) | 13/1516 (0.9%) | **OR 0.32** (0.11 to 0.98) | **6 fewer per 1,000** (from 8 fewer to 0 fewer) | ⨁◯◯◯ VERY LOW |  |
| **Length of stay (days) - RCT and cluster RCT** | | | | | | | | | | | | |
| 3 | randomised trials | serious ^a^ | serious ^b^ | not serious | serious ^c^ | none | 296 | 250 | - | MD **0.84 days lower** (2.59 lower to 0.9 higher) | ⨁◯◯◯ VERY LOW |  |
| **Length of stay (days) - Before-after studies** | | | | | | | | | | | | |
| 2 | observational studies | serious ^d^ | not serious | not serious | serious ^c^ | publication bias strongly suspected ^e^ | 1276 | 1089 | - | MD **0 days**  (0.43 lower to 0.44 higher) | ⨁◯◯◯ VERY LOW |  |

**CI:** Confidence interval; **OR:** Odds ratio; **MD:** Mean difference

#### Explanations

a. Some concerns in ROB2 in 2 studies

b. One is a cluster RCT

c. CI cross the clinical decision threshold

d. Moderate risk of bias in ROBINS -I

e. Suspected reporting bias (see ROBINS -I score)
