# Supplementary figures and images for: The impact of wearable continuous vital sign monitoring on deterioration detection and clinical outcomes in hospitalised patients: a systematic review and meta-analysis
Source: Crit Care. 2021 Sep 28;25:351. doi: 10.1186/s13054-021-03766-4 (PMC8477465; doi:10.1186/s13054-021-03766-4)

# Appendix 6 – Clinical Trial registry flowchart


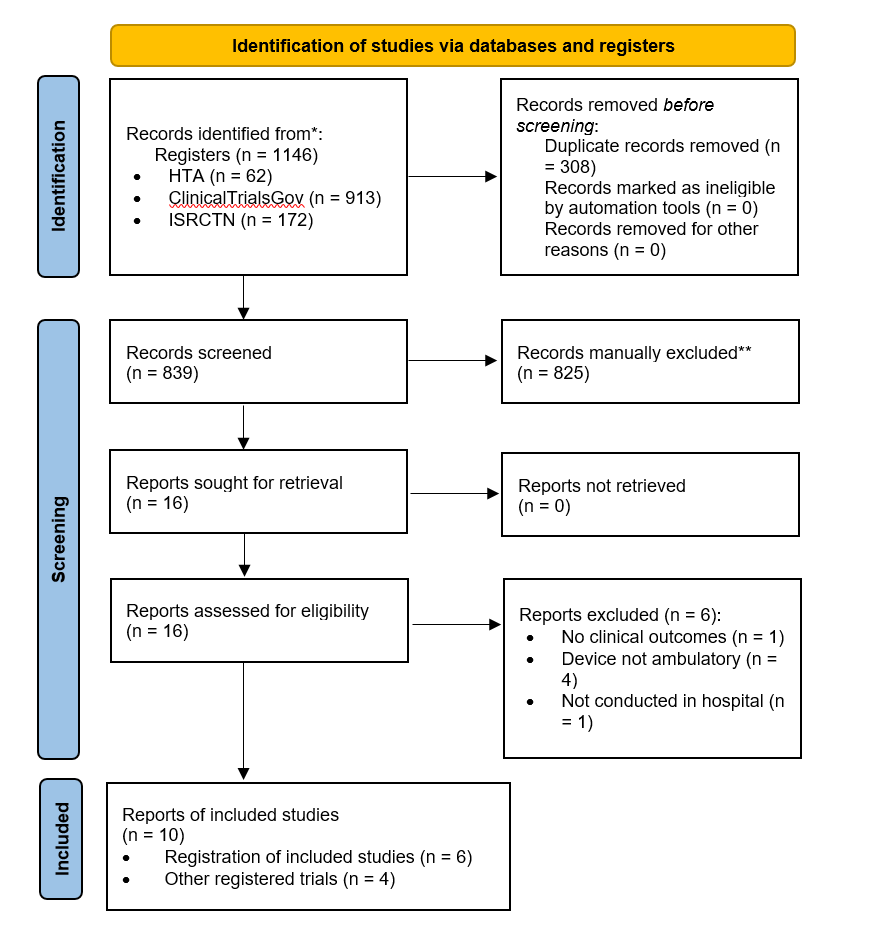

Supplement: Supplementary file 6 — Additional file 6. Clinical Trial registry flowchart [file 13054_2021_3766_MOESM6_ESM.docx]
