# Supplementary material for: The impact of wearable continuous vital sign monitoring on deterioration detection and clinical outcomes in hospitalised patients: a systematic review and meta-analysis
Source: Crit Care. 2021 Sep 28;25:351. doi: 10.1186/s13054-021-03766-4 (PMC8477465; doi:10.1186/s13054-021-03766-4)
Supplement: Supplementary file 7 — Additional file 7. Registered studies information [file 13054_2021_3766_MOESM7_ESM.docx]

# Appendix 7 – Registered studies information

| Study | Registration information | Outcomes | Reported? |
| --- | --- | --- | --- |
| Monsoon 2020 | **Registered:**  NCT03156608  (17^th^ May 2017, last update 1^st^ November 2017) [60]  **Published:**  19^th^ February 2020  [41] | **Primary:**  Amount of time in minutes with the interpretable fetal heart rate tracing during the course of labor | Yes |
|  |  | **Secondary:**  Quality and interpretability of the FHT in ten minute segments | Yes |
|  |  | Number and quality of uterine contractions in each 10 minute segment | Yes |
|  |  | Number of times fetal heart rate monitor requires adjusting | Yes |
|  |  | Need and reasons for additional monitoring devices such as change from Novii to standard monitoring devices or the use of intrauterine pressure catheter or fetal scalp electrode | Yes |
|  |  | Nursing time required for care | No |
|  |  | Overall cost of care | No |
|  |  | Maternal outcomes | Yes |
|  |  | Neonatal outcomes | Yes |
|  |  | Patient and provider satisfaction | Yes |
| Weenk 2020 & Weenk 2019 | **Registered:**  NCT02933307  (14^th^ October 2016, last update 25^th^ April 2017) [61]  **Published:**  15^th^ January 2019 [38]  10^th^ June 2020 [37] | **Primary:**  Expectations & experiences of patients | Yes |
|  |  | Expectations & experiences of care givers | Yes |
|  |  | **Secondary:**  MEWS scores based on continuous data and data measured by nurses | Yes |
|  |  | Amount of alarms by HealthPatch or ViSi Mobile | Yes |
|  |  | Time between alarm (continuous data) and next regular MEWS measurement (nurse) | Yes |
|  |  | Amount of extra MEWS measurements by nurses due to alarms | Yes |
|  |  | Admission to ICU | No |
|  |  | Duration of ICU hospitalization in days | No |
|  |  | Complications caused by disease or surgical procedure | No |
|  |  | Adverse events caused by devices | Yes |
|  |  | Technical failures of devices | Yes |
|  |  | Outcomes of the State Trait Anxiety Inventory (STAI) | Yes |
|  |  | Outcomes of the Pain Catastrophizing Scale (PCS) | Yes |
|  |  | System usability Scale | Yes |
| Downey 2018 | **Registered:**  ISRCTN60999823  (Retrospectively registered, 15^th^ December 2016, last update 13^th^ December 2018) [62]  **Published:**  11^th^ December 2018 [29] | **Primary:**  Time to antibiotics in patients who are diagnosed as suffering from sepsis | Yes |
|  |  | **Secondary:**  In-hospital mortality | Yes |
|  |  | Length of hospital stay | Yes |
|  |  | Number of admissions to Level II/III care | Yes |
|  |  | Length of stay at Level II/III | No |
|  |  | Patient satisfaction | Yes [51] |
|  |  | Staff satisfaction | Yes [51] |
| Downey 2020 | **Registered:**  ISRCTN16601772 (Prospectively registered, 3^rd^ July 2017, last update 4^th^ October 2018) [63]  **Published:**  23^rd^ November 2020 [30] | **Primary:**  Recruitment rate | Yes |
|  |  | Adherence to protocol | Yes |
|  |  | Amount of missing data | Yes |
|  |  | Optimal outcome measures to test effectiveness | Yes |
|  |  | Estimation of sample size for definitive RCT | Yes |
|  |  | **Secondary:**  Time to antibiotics in cases of sepsis | Yes |
|  |  | Number of HDU/ICU admissions | Yes |
|  |  | Length of stay in HDU/ICU | Yes |
|  |  | Total length of stay in hospital | Yes |
|  |  | Number of postoperative complications | Yes |
|  |  | Number of re-interventions | Yes |
|  |  | Patient acceptability | Yes |
|  |  | Nursing acceptability | No |
|  |  | 30-day readmission rate | Yes |
| Skraastad 2019 | **Registered:**  NCT03438578  (19^th^ February 2018, last update 24^th^ September 2020) [64]  **Published:**  14^th^ March 2019 [27] | **Primary:**  Time to Mobilization | Yes |
|  |  | Pain Measurement | Yes |
|  |  | Patient Satisfaction | Yes |
| Watkinson 2020 | **Registered:**  ISRCTN58660550  (Retrospectively registered, 8^th^ June 2017, last update 26^th^ November 2020) [43]  **Published (Pre-print):**  2^nd^ December 2020 [40] | **Primary:**  Length of stay | Yes |
|  |  | **Secondary:**  Mortality | Yes |
|  |  | Unplanned ICU admission | Yes |
|  |  | Clinical deteriorations | Yes |
|  |  | Sensitivity, specificity, positive and negative prediction | No |
| Kisner 2009 | **Not registered** |  |  |
| Verrillo 2018 | **Not registered** |  |  |
| Weller 2018 | **Not registered** |  |  |
