# Supplementary material for: The impact of wearable continuous vital sign monitoring on deterioration detection and clinical outcomes in hospitalised patients: a systematic review and meta-analysis
Source: Crit Care. 2021 Sep 28;25:351. doi: 10.1186/s13054-021-03766-4 (PMC8477465; doi:10.1186/s13054-021-03766-4)
Supplement: Supplementary file 8 — Additional file 8. Quality assessment scales [file 13054_2021_3766_MOESM8_ESM.docx]

# Appendix 8 – Quality assessment scales

## Non randomised studies

### NOS scale

| Study | Selection | Comparability | Outcome | Total |
| --- | --- | --- | --- | --- |
| Weller et al, 2018 | **** | ** | *** | 9 |
| Verrillo et al, 2018 | **** | ** | *** | 9 |
| Kisner et al, 2009 | **** | ** | *** | 9 |
| Watkinson et al 2020 | **** | ** | *** | 9 |

### ROBINS-I scale

| Study | 1. Confounding | 2. Participant selection | 3. Classification of interventions | 4. Deviations from intended interventions | 5. Missing data | 6. Measurement of outcomes | 7. Selection of reported result | Overall Risk of bias |
| --- | --- | --- | --- | --- | --- | --- | --- | --- |
| Kisner et al, 2009 | Low risk  (Unpredictable) | Moderate  (Unpredictable) | Low risk  (Unpredictable) | Low risk  (Unpredictable) | Low risk  (Unpredictable) | Moderate  (Favours experimental) | Moderate  (Unpredictable) | Moderate  (Unpredictable) |
| Weller et al. 2018 | Low risk  (Unpredictable) | Moderate  (Unpredictable) | Low risk  (Unpredictable) | Low risk  (Unpredictable) | Low risk  (Unpredictable) | Low risk  (Unpredictable) | Moderate  (Unpredictable) | Moderate  (Unpredictable) |
| Verrillo et al. 2018 | Low risk  (Unpredictable) | Low risk  (Unpredictable) | Low risk  (Unpredictable) | Low risk  (Unpredictable) | Low risk  (Unpredictable) | Moderate  ( Unpredictable ) | Moderate  (Unpredictable) | Moderate  (Unpredictable) |
| Watkinson et al. 2020 | Low risk  (Unpredictable) | Moderate  (Unpredictable) | Low risk  (Unpredictable) | Low risk  (Unpredictable) | Low risk  (Unpredictable) | Low risk  (Unpredictable) | Low risk  (Unpredictable) | Moderate  (Unpredictable) |

## Randomised studies

### ROB2

| Study | 1a. Randomization | 1b. Cluster randomization timing | 2. Effect of assignment to intervention | 3. Missing outcome data | 4. Measurement of the outcome | 5. Selection of the reported result | Overall Risk of bias |
| --- | --- | --- | --- | --- | --- | --- | --- |
| Downey et al, 2018 | Some concerns  (Unpredictable) | Low risk  (Unpredictable) | Low risk  (Unpredictable) | Low risk  (Unpredictable) | Low risk  (Unpredictable) | Low risk  (Unpredictable) | Some concerns  (Unpredictable) |
| Skraastad et al 2019 | Low risk  (Unpredictable) | N/A | Some concerns  (Unpredictable) | Low risk  (Unpredictable) | Some concerns  (Unpredictable) | Low risk  (Unpredictable) | Some concerns  (Unpredictable) |
| Downey et al, 2020 | Low risk  (Unpredictable) | N/A | Low risk  (Unpredictable) | Low risk  (Unpredictable) | Low risk  (Unpredictable) | Low risk  (Unpredictable) | Low risk  (Unpredictable) |
| Monsoon et al. 2020 | Low risk  (Unpredictable) | N/A | Low risk  (Unpredictable) | Low risk  (Unpredictable) | Low risk  (Unpredictable) | Low risk  (Unpredictable) | Low risk  (Unpredictable) |
| Weenk et al, 2019 & 2020 | Some concerns  (Unpredictable) | N/A | Low risk  (Unpredictable) | Low risk  (Unpredictable) | Low risk  (Unpredictable) | Low risk  (Unpredictable) | Some concerns  (Unpredictable) |

## MMAT – all studies

| **Category of study designs** | **Methodological quality criteria** | **Weller 2017** | **Verrillo 2018** | **Downey 2018** | **Skraastad 2019** | **Kisner 2009** | **Downey 2020** | **Watkinson 2020** | **Weenk 2019&2020** | **Monsoon 2020** |
| --- | --- | --- | --- | --- | --- | --- | --- | --- | --- | --- |
|  |  | Cohort study | Cohort study | Cluster RCT | RCT | Retrospective | RCT | Cohort study | RCT | RCT |
| Screening questions  (for all types) | S1. Are there clear research questions? | Y | Y | Y | Y | Y | Y | Y | Y | Y |
|  | S2. Do the collected data allow to address the research questions? | Y | Y | Y | Y | Y | Y | Y | Y | Y |
| 1. Qualitative | 1.1. Is the qualitative approach appropriate to answer the research question? |  |  |  |  |  |  |  |  |  |
|  | 1.2. Are the qualitative data collection methods adequate to address the research question? |  |  |  |  |  |  |  |  |  |
|  | 1.3. Are the findings adequately derived from the data? |  |  |  |  |  |  |  |  |  |
|  | 1.4. Is the interpretation of results sufficiently substantiated by data? |  |  |  |  |  |  |  |  |  |
|  | 1.5. Is there coherence between qualitative data sources, collection, analysis and interpretation? |  |  |  |  |  |  |  |  |  |
| 2. Quantitative randomized controlled trials | 2.1. Is randomization appropriately performed? |  |  | N | Y |  | Y |  | Y | Y |
|  | 2.2. Are the groups comparable at baseline? |  |  | Y | Y |  | Y |  | Y | Y |
|  | 2.3. Are there complete outcome data? |  |  | Y | Y |  | Y |  | Y | Y |
|  | 2.4. Are outcome assessors blinded to the intervention provided? |  |  | N | N |  | N |  | Y | Y |
|  | 2.5 Did the participants adhere to the assigned intervention? |  |  | Y | Y |  | Y |  | Y | Y |
| 3. Quantitative non-randomized | 3.1. Are the participants representative of the target population? | Y | Y |  |  | Y |  | Y |  |  |
|  | 3.2. Are measurements appropriate regarding both the outcome and intervention (or exposure)? | Y | Y |  |  | Y |  | Y |  |  |
|  | 3.3. Are there complete outcome data? | Y | Y |  |  | Y |  | Y |  |  |
|  | 3.4. Are the confounders accounted for in the design and analysis? | Y | Y |  |  | Y |  | Y |  |  |
|  | 3.5. During the study period, is the intervention administered (or exposure occurred) as intended? | Y | Y |  |  | Y |  | Y |  |  |
| 4. Quantitative descriptive | 4.1. Is the sampling strategy relevant to address the research question? |  |  |  |  |  |  |  |  |  |
|  | 4.2. Is the sample representative of the target population? |  |  |  |  |  |  |  |  |  |
|  | 4.3. Are the measurements appropriate? |  |  |  |  |  |  |  |  |  |
|  | 4.4. Is the risk of nonresponse bias low? |  |  |  |  |  |  |  |  |  |
|  | 4.5. Is the statistical analysis appropriate to answer the research question? |  |  |  |  |  |  |  |  |  |
| 5. Mixed methods | 5.1. Is there an adequate rationale for using a mixed methods design to address the research question? |  |  |  |  |  |  |  |  |  |
|  | 5.2. Are the different components of the study effectively integrated to answer the research question? |  |  |  |  |  |  |  |  |  |
|  | 5.3. Are the outputs of the integration of qualitative and quantitative components adequately interpreted? |  |  |  |  |  |  |  |  |  |
|  | 5.4. Are divergences and inconsistencies between quantitative and qualitative results adequately addressed? |  |  |  |  |  |  |  |  |  |
|  | 5.5. Do the different components of the study adhere to the quality criteria of each tradition of the methods involved? |  |  |  |  |  |  |  |  |  |
